# Supplementary material for: An engineered CRISPR-Cas12a variant and DNA-RNA hybrid guides enable robust and rapid COVID-19 testing
Source: Nat Commun. 2021 Mar 19;12:1739. doi: 10.1038/s41467-021-21996-6 (PMC7979722; doi:10.1038/s41467-021-21996-6)
Supplement: Supplementary file 1 — Description of Additional Supplementary Files [file 41467_2021_21996_MOESM1_ESM.pdf]

File Name: Supplementary Data 1
Description: Compilation of CRISPR-based diagnostic assays for COVID-19 (as of 18 December 2020)

File Name: Supplementary Data 2
Description: Alignment of each gRNA against all the sequenced SARS-CoV-2 genomes annotated in GISAID (as of 17 June 2020)

File Name: Supplementary Data 3
Description: Cost of VaNGuard test using either a fluorescent readout or a lateral flow visual readout

File Name: Supplementary Data 4
Description: Sequences of all oligonucleotides used in our study
