# Supplementary material for: An engineered CRISPR-Cas12a variant and DNA-RNA hybrid guides enable robust and rapid COVID-19 testing
Source: Nat Commun. 2021 Mar 19;12:1739. doi: 10.1038/s41467-021-21996-6 (PMC7979722; doi:10.1038/s41467-021-21996-6)
Supplement: Supplementary file 2 — Supplementary Information [file 41467_2021_21996_MOESM2_ESM.pdf]

Supplementary Information

An engineered CRISPR-Cas12a variant and DNA-RNA hybrid guides enable robust and rapid COVID-19 testing

Kean Hean Ooi[1,2,8], Mengying Mandy Liu[1,2,8], Jie Wen Douglas Tay[1,2,3,8], Seok Yee Teo[1,2,3,8], Pornchai Kaewsapsak[2,8], Shengyang Jin[3], Chun Kiat Lee[4], Jingwen Hou[5], Sebastian Maurer-Stroh[6], Weisi Lin[5], Benedict Yan[4], Gabriel Yan[7], Yong-Gui Gao[3], Meng How Tan[1,2]

[1]School of Chemical and Biomedical Engineering, Nanyang Technological University, Singapore 637459, Singapore

[2]Genome Institute of Singapore, Agency for Science Technology and Research, Singapore 138672, Singapore

[3]School of Biological Sciences, Nanyang Technological University, Singapore 637551, Singapore

[4]Department of Laboratory Medicine, National University Hospital, National University Health System, Singapore 119074, Singapore

[5]School of Computer Science and Engineering, Nanyang Technological University, Singapore 639798, Singapore

[6]Bioinformatics Institute, Agency for Science Technology and Research, Singapore 138671, Singapore

[7]Division of Infectious Diseases, Department of Medicine, National University Hospital, National University Health System, Singapore 119228, Singapore

[8]These authors contributed equally.

[*]Correspondence: mh.tan@ntu.edu.sg or tanmh@gis.a-star.edu.sg

SUPPLEMENTARY FIGURES

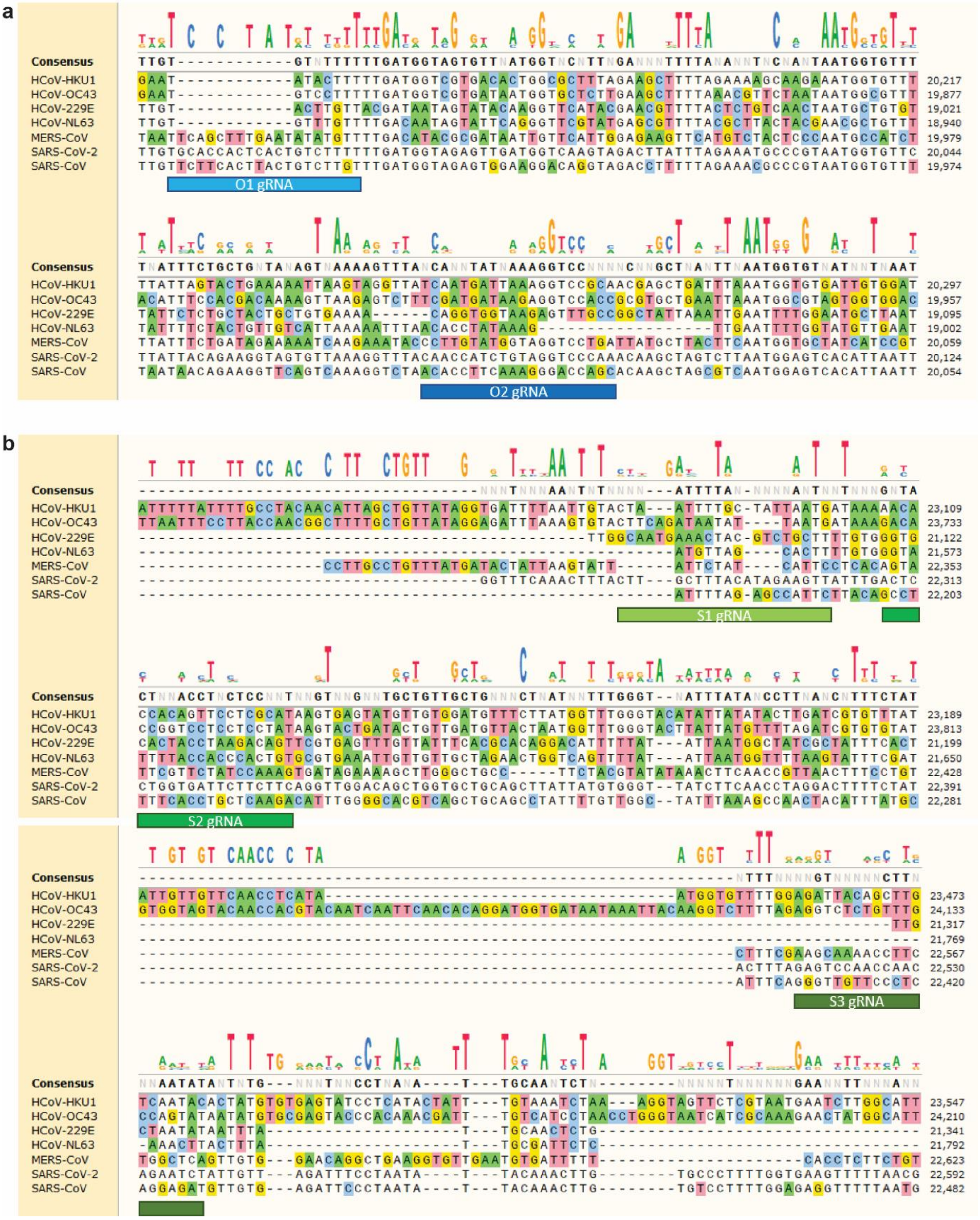

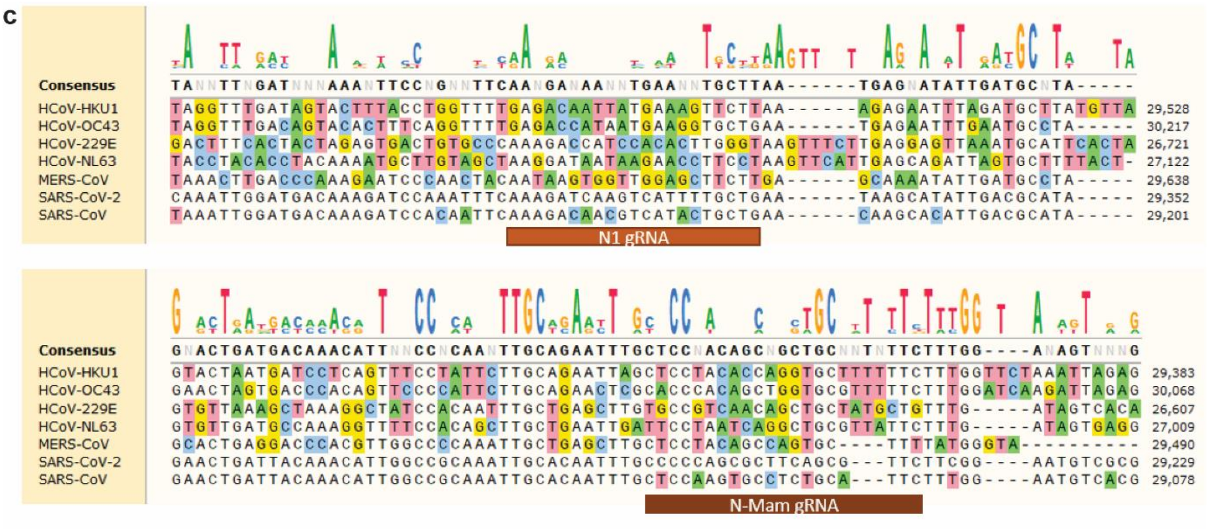

Supplementary Fig. 1 Multiple sequence alignment between seven coronaviruses, including SARS-CoV-2, showing the region of the (**a**) ORF1b gene, (**b**) S-gene, and (**c**) N-gene targeted by the gRNAs that we evaluated with different Cas12a nucleases in our study. The TTTV PAM precedes each 20nt spacer.

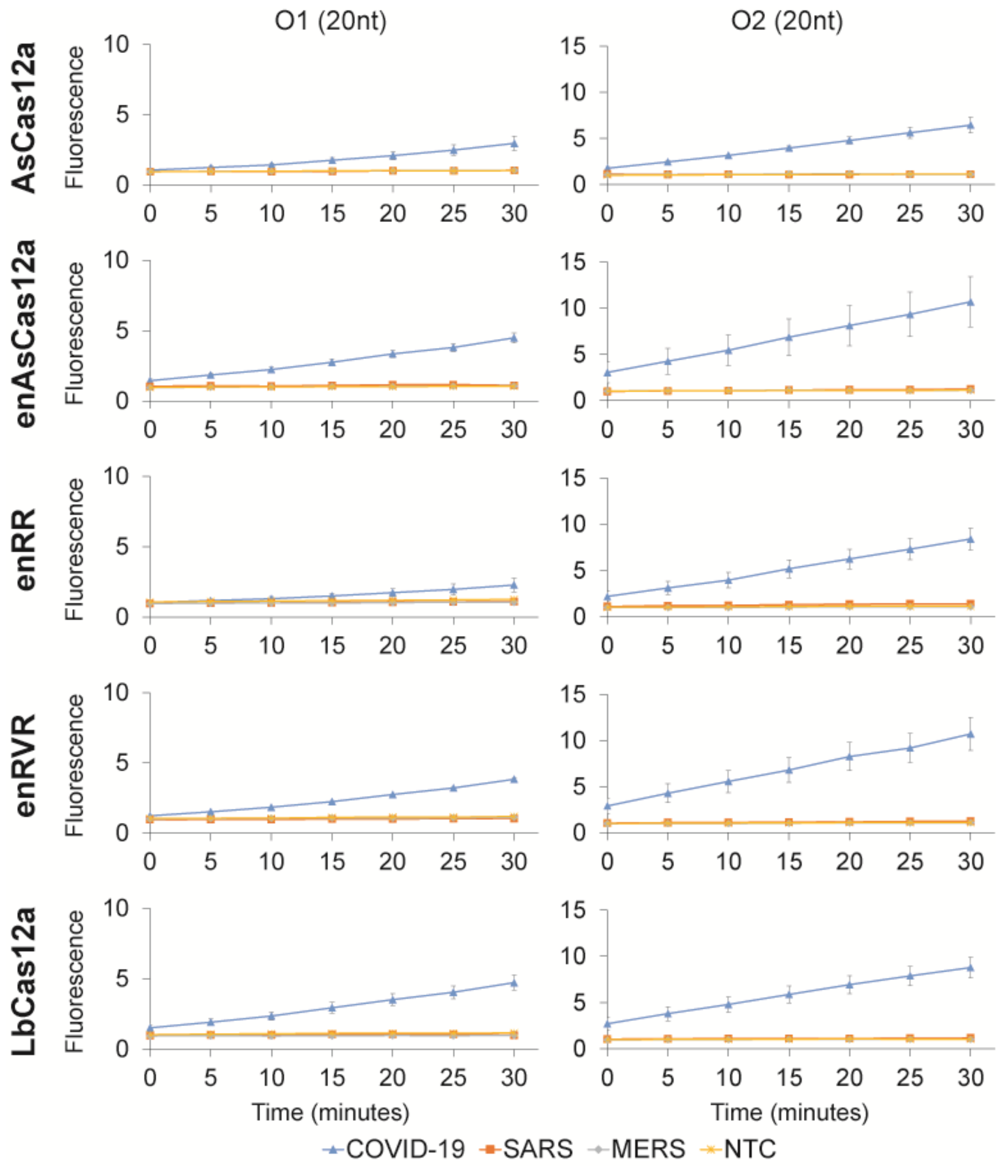

O1 (20nt)
O2 (20nt)
AsCas12a
enAsCas12a
enRR
enRVR
LbCas12a
Fluorescence
Time (minutes)
COVID-19
SARS
MERS
NTC

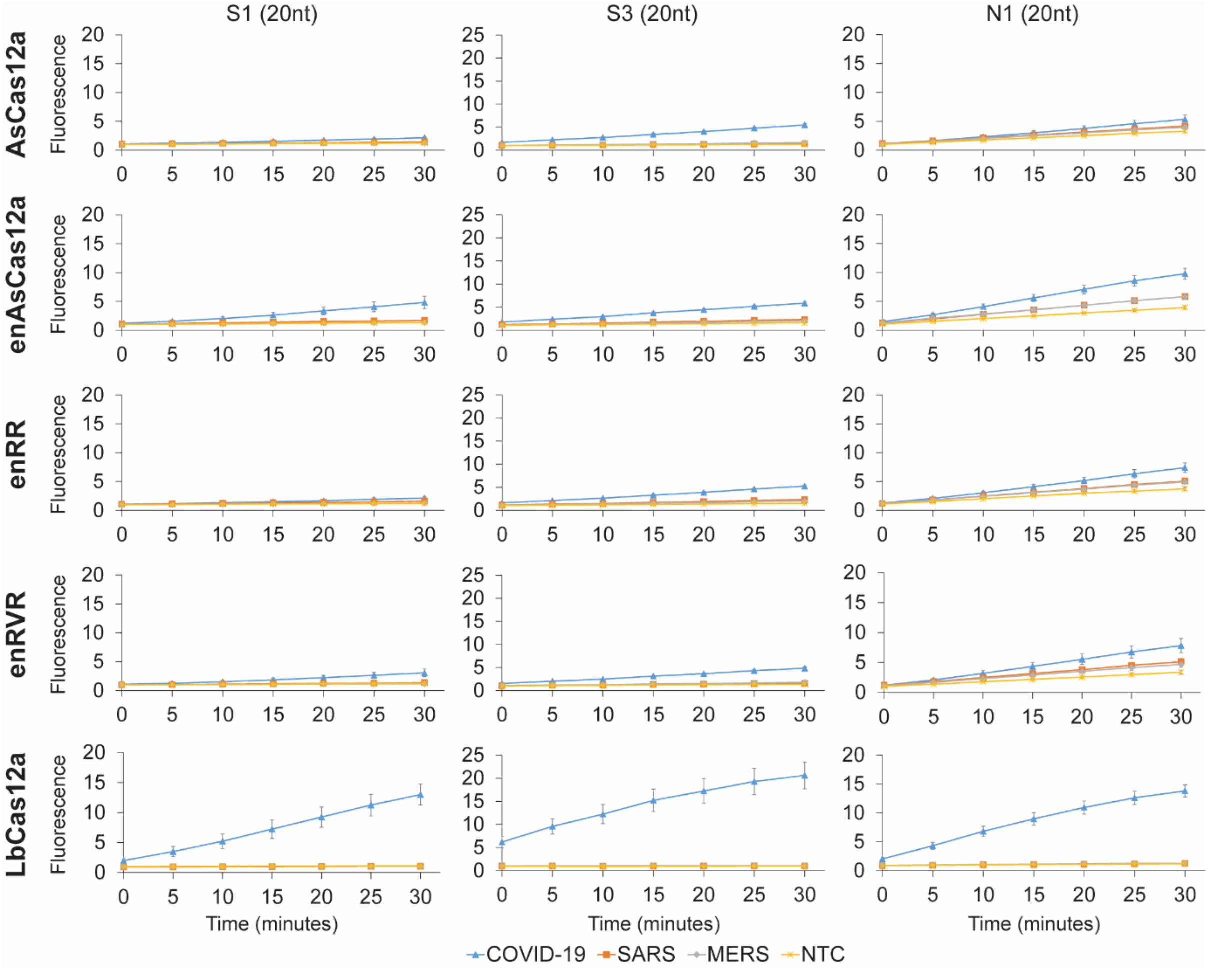

Supplementary Fig. 2 Time courses of the fluorescence intensity in our trans-cleavage assays for various Cas12a nucleases complexed with perfect matched (PM) gRNAs targeting the O1, O2, S1, S3, and N1 loci of the SARS-CoV-2 genome. The assays were performed at 24°C and approximately 1E11 copies of purified DNA template were used as input. All the measurements were normalized to the no-template control (NTC) at the start of the experiment. Data represent mean ± s.e.m. (n = 3 [O1, O2, S3], 4 [S1], or 6 [N1] biological replicates).

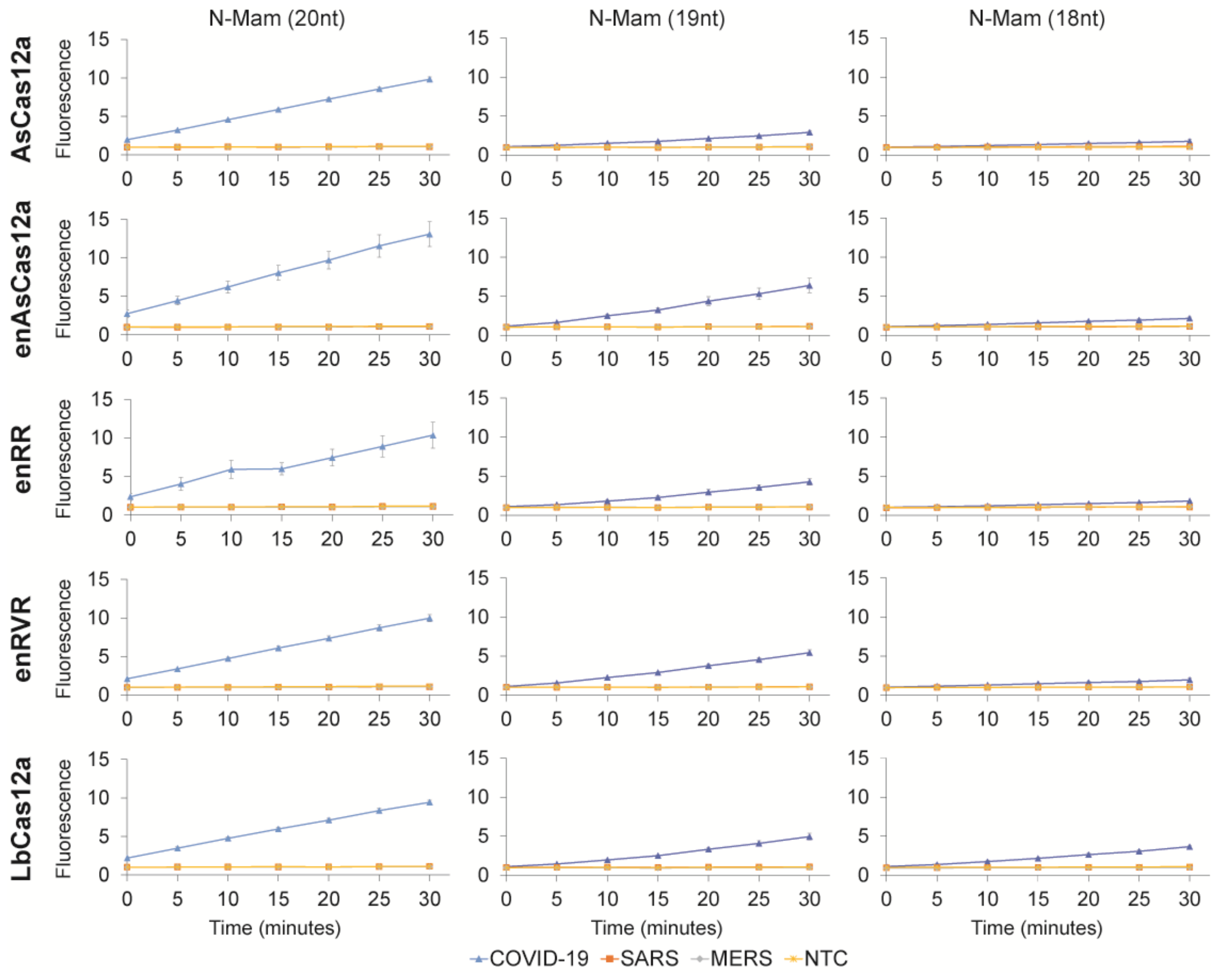

Supplementary Fig. 3 Time courses of the fluorescence intensity in our trans-cleavage assays for various Cas12a nucleases complexed with perfect matched (PM) N-Mam gRNAs of three different spacer lengths. The assays were performed at 24°C and 1E11 copies of purified DNA template were used as input. All the measurements were normalized to the no-template control (NTC) at the start of the experiment. Data represent mean ± s.e.m. (n = 3 biological replicates).

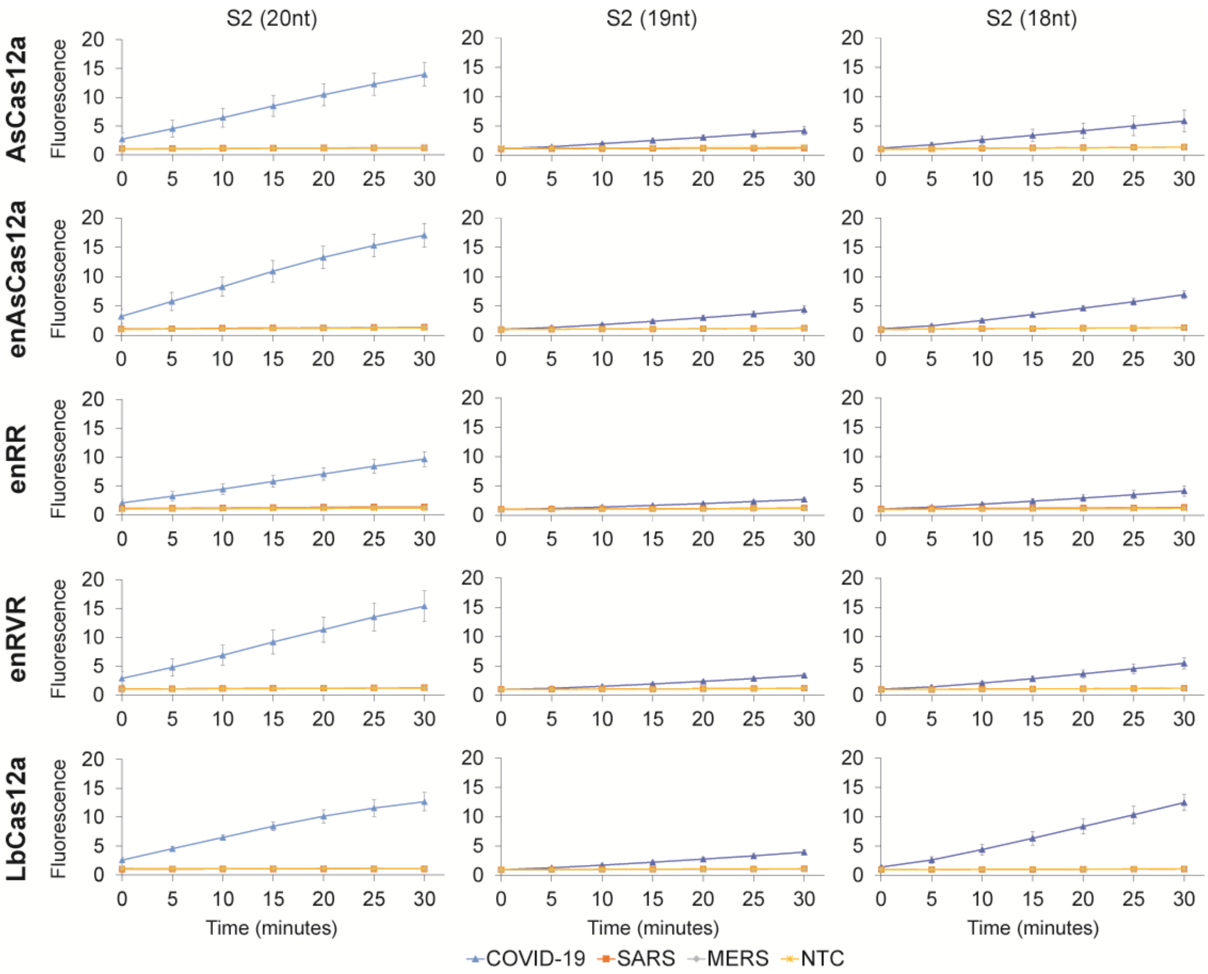

Supplementary Fig. 4 Time courses of the fluorescence intensity in our trans-cleavage assays for various Cas12a nucleases complexed with perfect matched (PM) S2 gRNAs of three different spacer lengths. The assays were performed at 24°C and 1E11 copies of purified DNA template were used as input. All the measurements were normalized to the no-template control (NTC) at the 0min timepoint. Data represent mean ± s.e.m. (n = 3 [20nt] or 4 [18nt, 19nt] biological replicates).

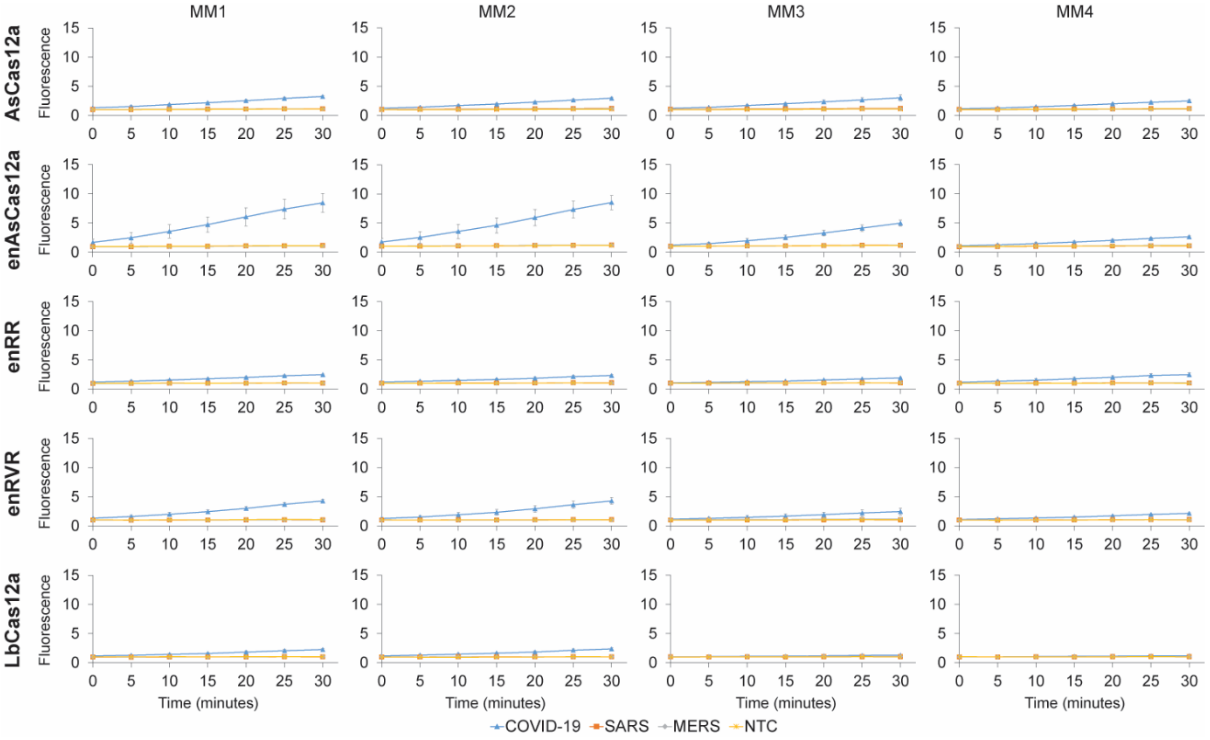
MM1
MM2
MM3
MM4
AsCas12a
enAsCas12a
enRR
enRVR
LbCas12a
Fluorescence
Time (minutes)
COVID-19
SARS
MERS
NTC

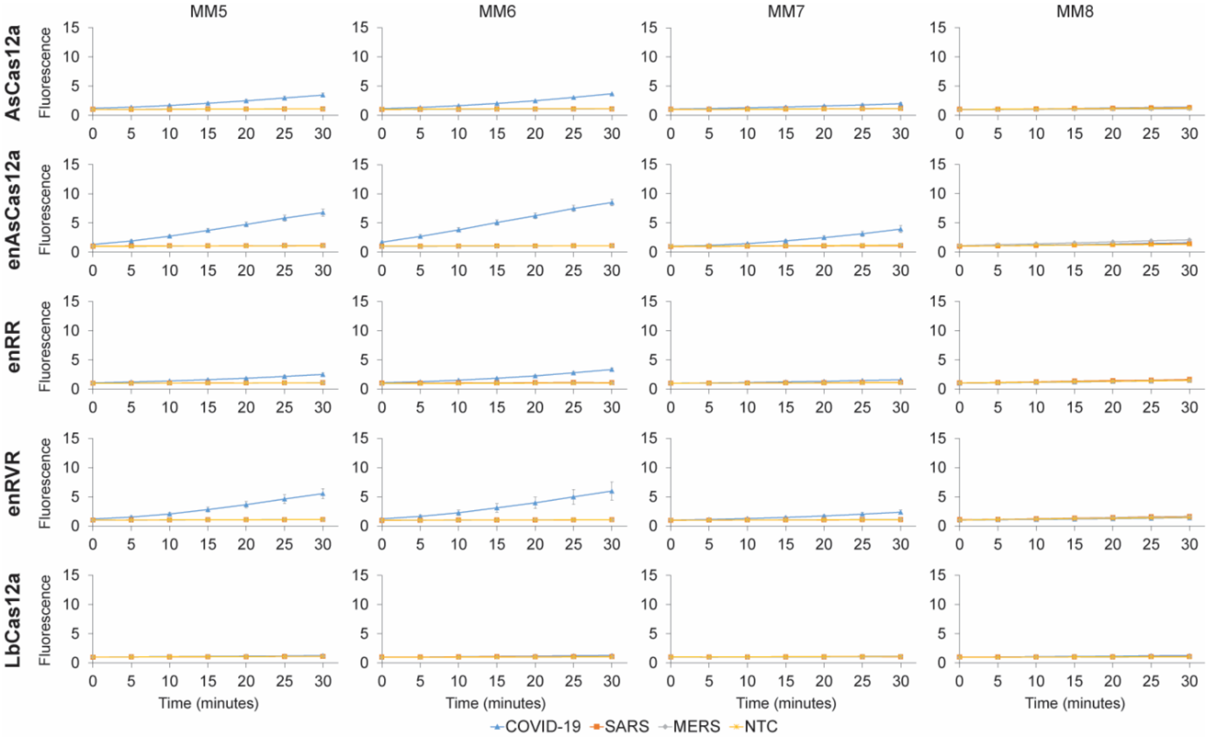
MM5
MM6
MM7
MM8
AsCas12a
enAsCas12a
enRR
enRVR
LbCas12a
Fluorescence
Time (minutes)
COVID-19
SARS
MERS
NTC

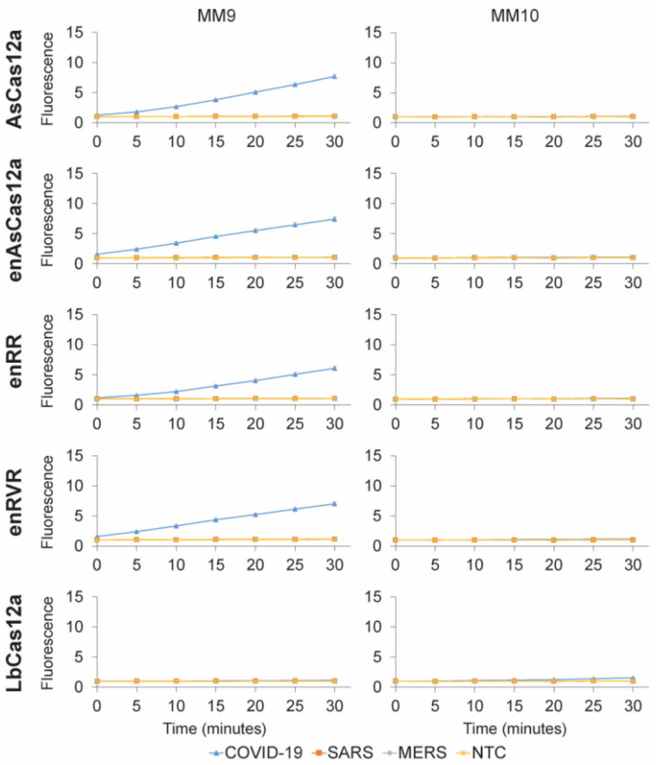

Supplementary Fig. 5 Time courses of the fluorescence intensity in our trans-cleavage assays for various Cas12a nucleases complexed with mismatched (MM) N-Mam gRNAs of spacer length 20nt. The assays were performed at 24ºC and 1E11 copies of purified DNA template were used as input. Data represent mean ± s.e.m. (n = 3 [AsCas12a for MM5, MM6, MM8, MM9, and MM10; enAsCas12a; enRR; enRVR; LbCas12a] or 4 [AsCas12a for MM1, MM2, MM3, MM4, and MM7] biological replicates).

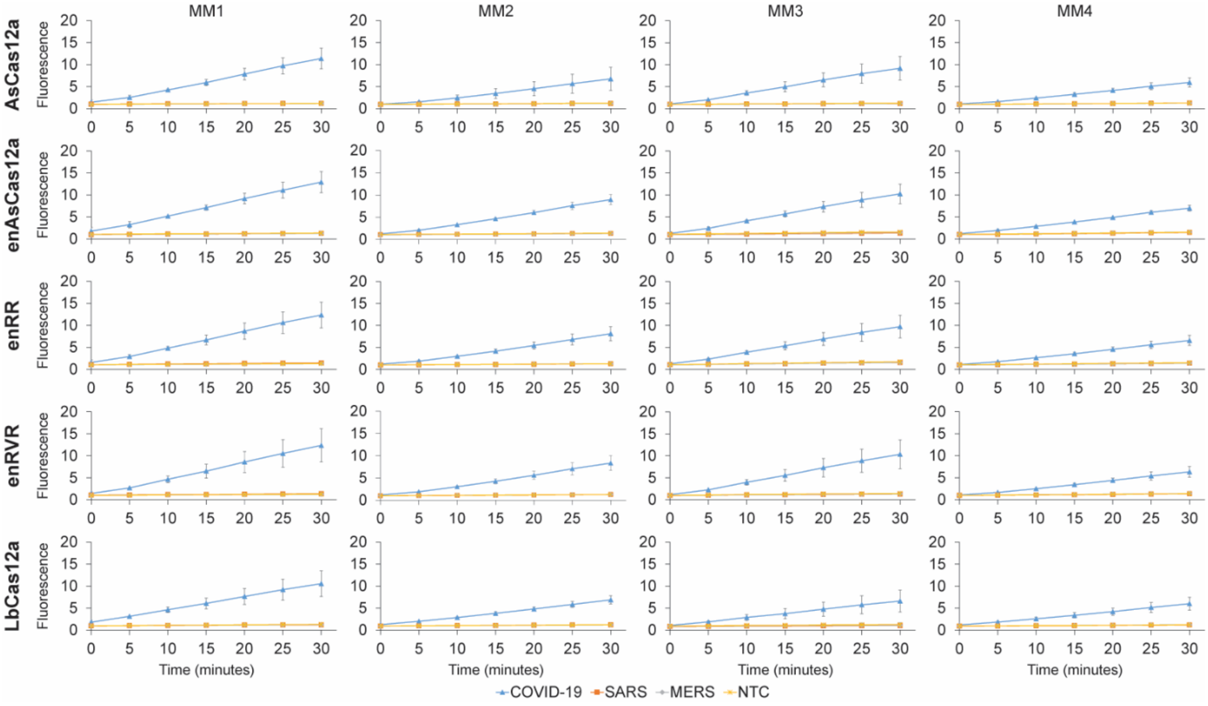
MM1
MM2
MM3
MM4
AsCas12a
enAsCas12a
enRR
enRVR
LbCas12a
Fluorescence
Time (minutes)
COVID-19
SARS
MERS
NTC

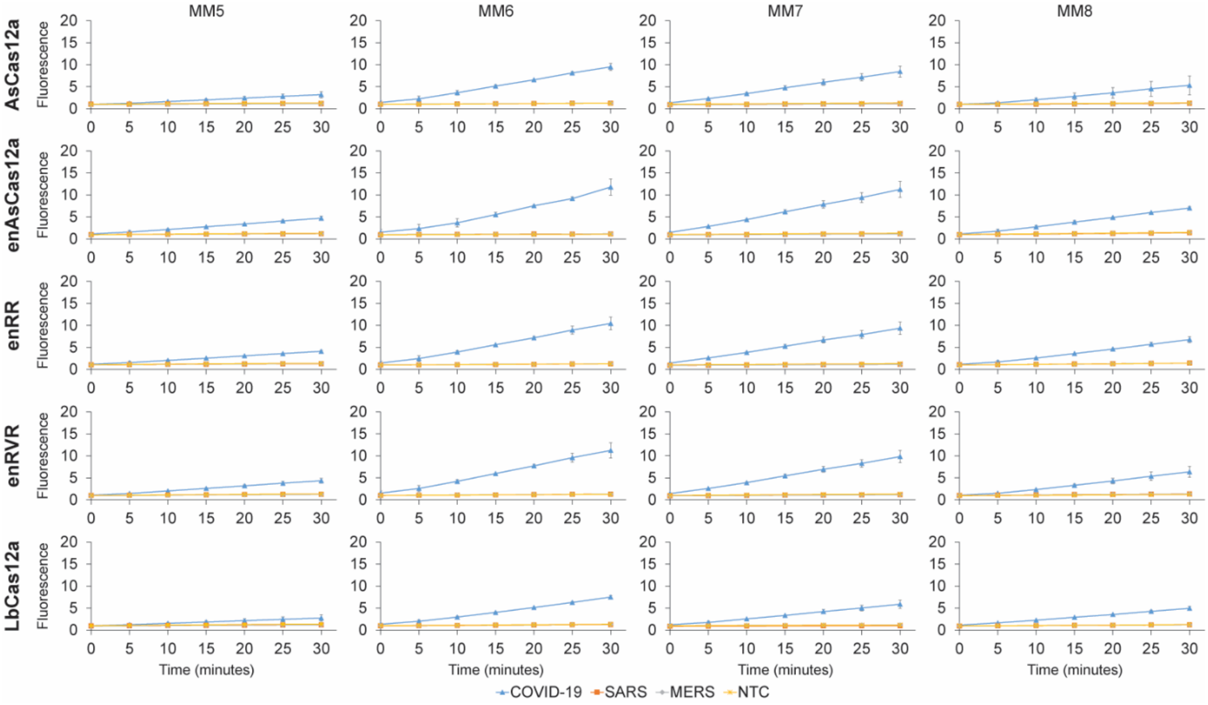
MM5
MM6
MM7
MM8
AsCas12a
enAsCas12a
enRR
enRVR
LbCas12a
Fluorescence
Time (minutes)
COVID-19
SARS
MERS
NTC

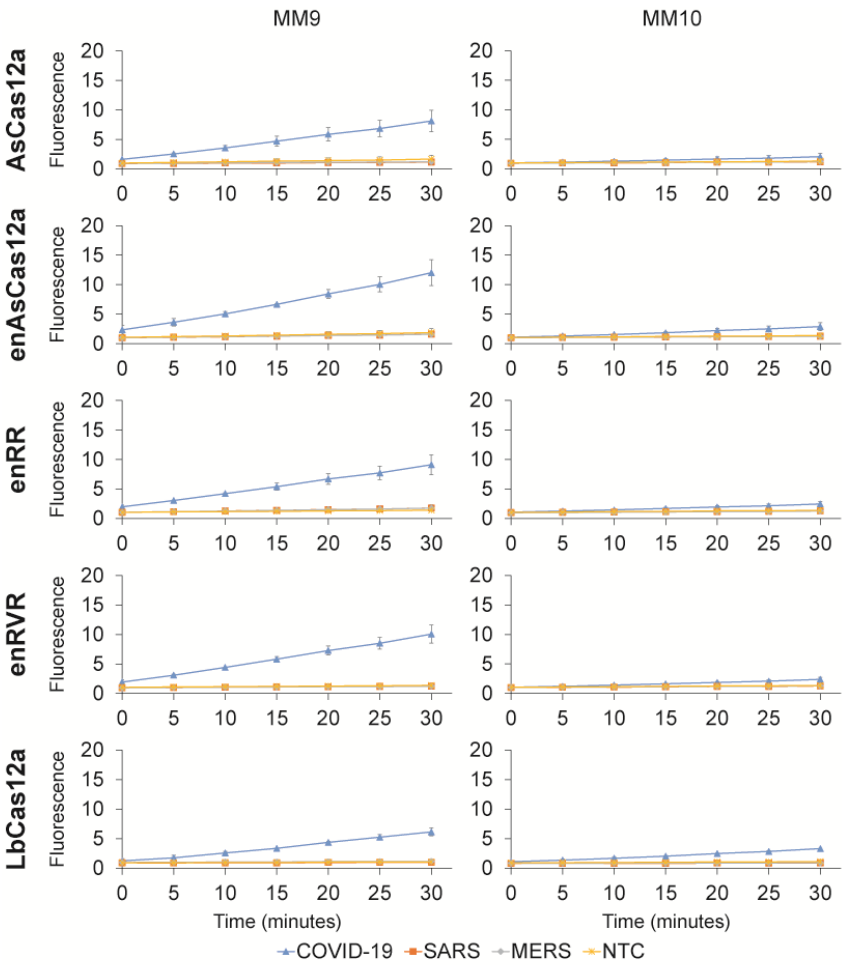

Supplementary Fig. 6 Time courses of the fluorescence intensity in our trans-cleavage assays for various Cas12a nucleases complexed with mismatched (MM) S2 gRNAs of spacer length 20nt. The assays were performed at 24°C and 1E11 copies of purified DNA template were used as input. Data represent mean ± s.e.m. (n = 2 biological replicates).

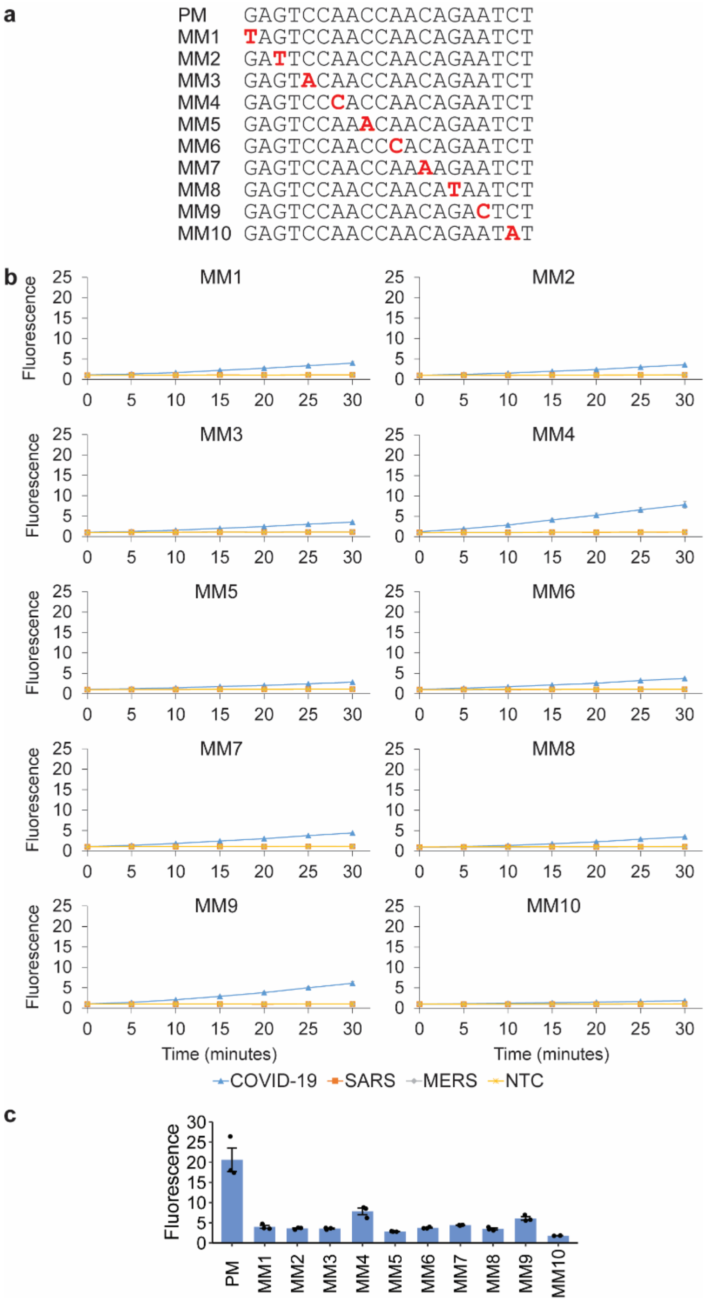

Supplementary Fig. 7 Mismatch tolerance of LbCas12a at the S3 locus.

a Sequences of perfect matched (PM) or mismatched (MM) spacers targeting the S3 locus. Each mismatched position is indicated by a bold red letter.

b Time courses of the fluorescence intensity in our trans-cleavage assays for LbCas12a complexed with S3 MM gRNAs. The assays were performed at 24°C and 1E11 copies of DNA template were used as input. Data represent mean ± s.e.m. (n = 3 biological replicates).

c Summary of LbCas12a's collateral activity when complexed with a S3 PM or MM gRNA. The fluorescence measurements here were taken after 30 minutes of cleavage reaction using a microplate reader and all the readings were normalized to the NTC at the start of the experiment. Data represent mean ± s.e.m. (n = 3 biological replicates).

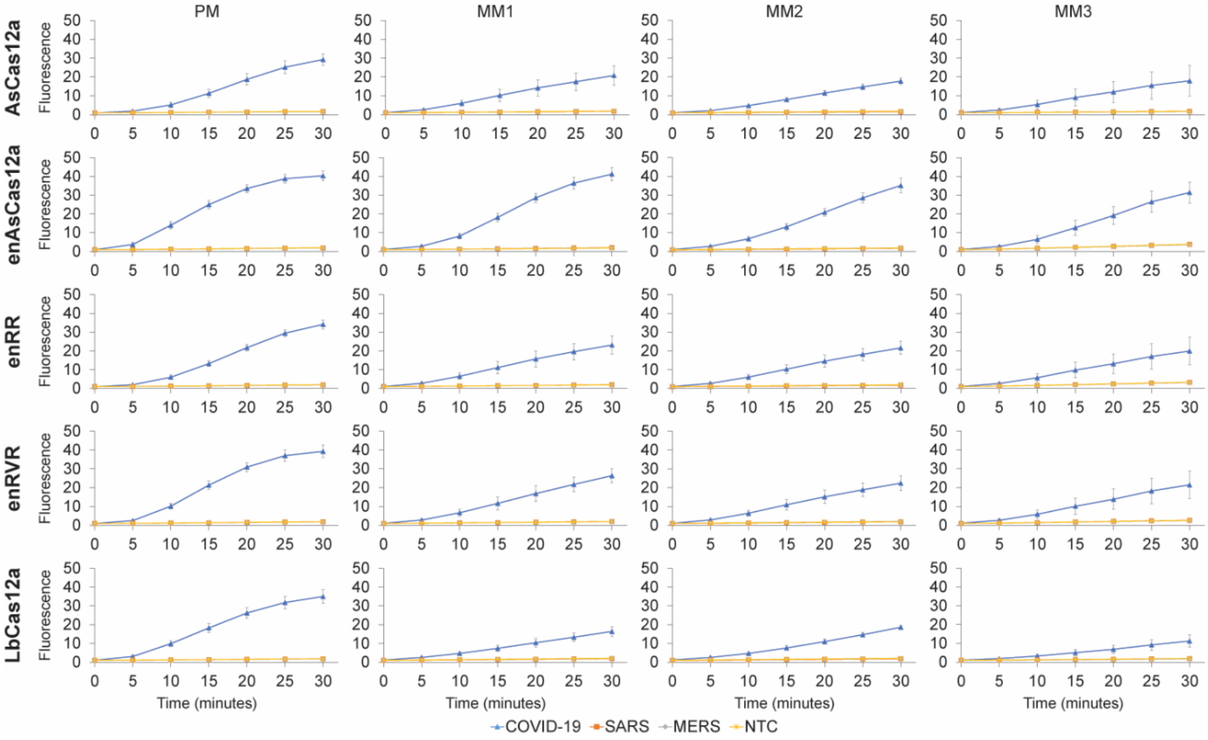
PM
MM1
MM2
MM3
AsCas12a
enAsCas12a
enRR
enRVR
LbCas12a
Fluorescence
Time (minutes)
COVID-19
SARS
MERS
NTC

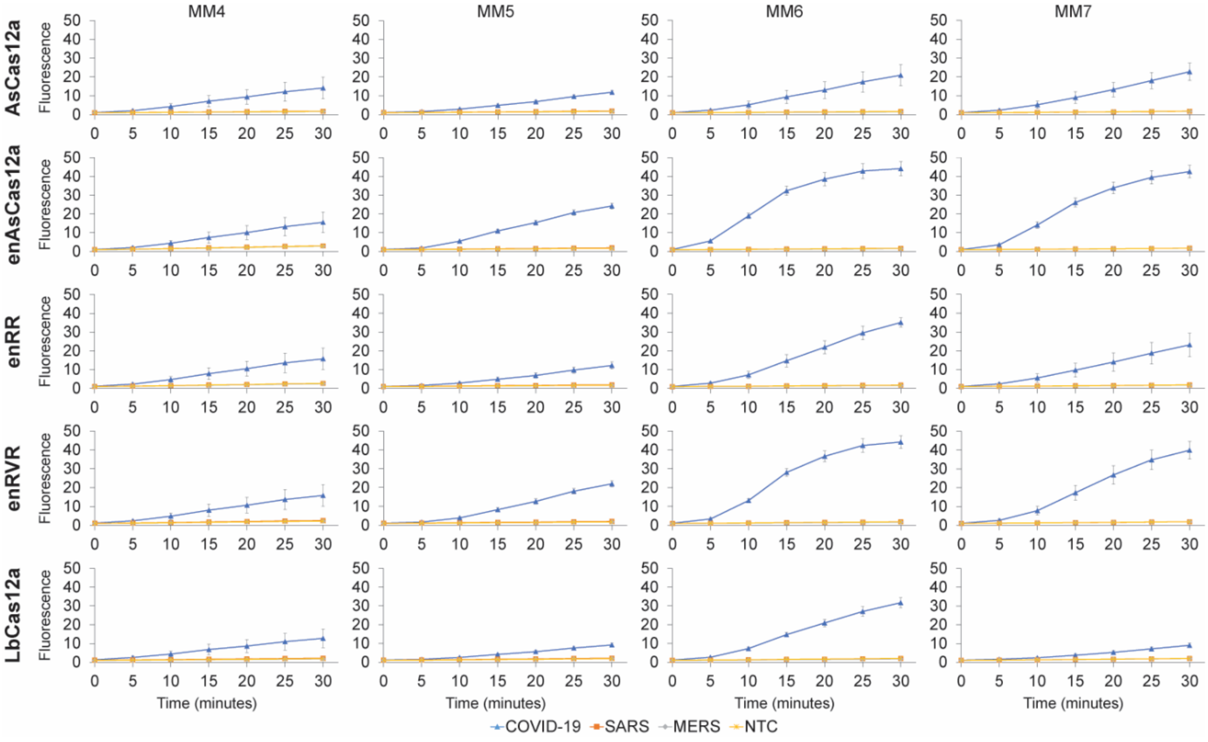
MM4
MM5
MM6
MM7
AsCas12a
enAsCas12a
enRR
enRVR
LbCas12a
Fluorescence
Time (minutes)
COVID-19
SARS
MERS
NTC

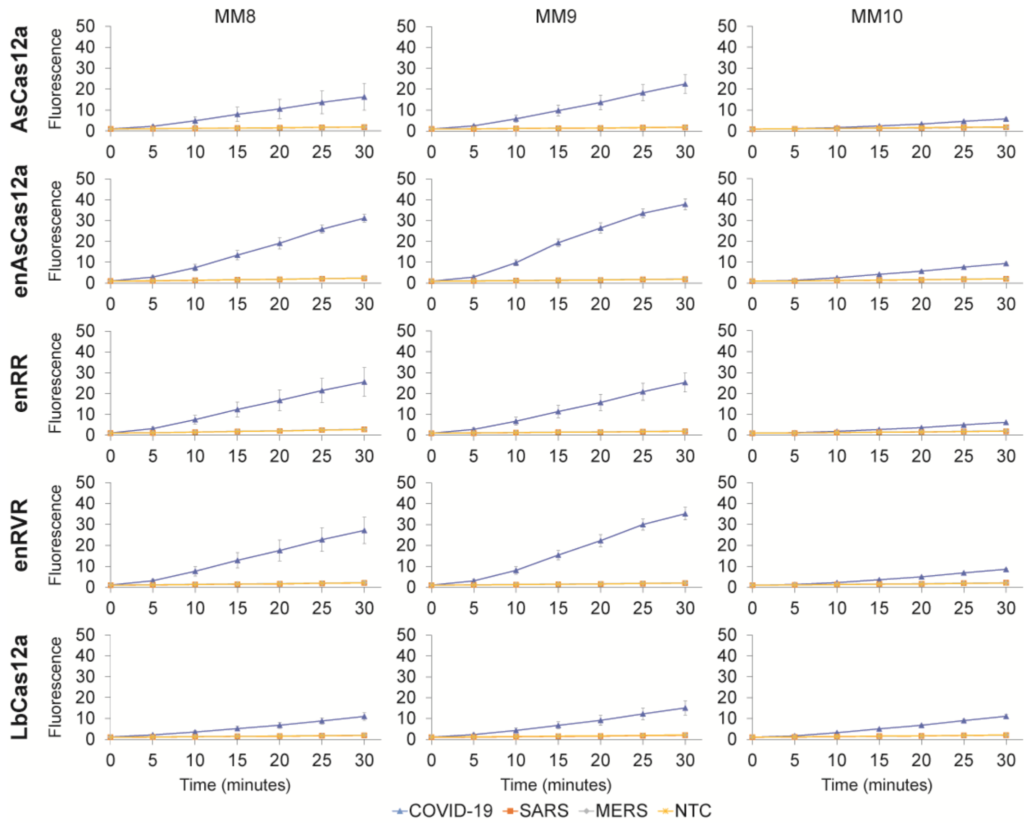

Supplementary Fig. 8 Time courses of the fluorescence intensity in our trans-cleavage assays for various Cas12a nucleases complexed with either perfect matched (PM) or mismatched (MM) S2 gRNAs of spacer length 20nt. The assays were performed at 37°C and 1E11 copies of purified DNA template were used as input. All the measurements were normalized to the no-template control (NTC) at the 0min timepoint. We observed that when the Cas detection reaction was performed at 37°C, it proceeded around twice as fast as the same reaction at 24°C (room temperature). Data represent mean ± s.e.m. (n = 3 biological replicates).

S4

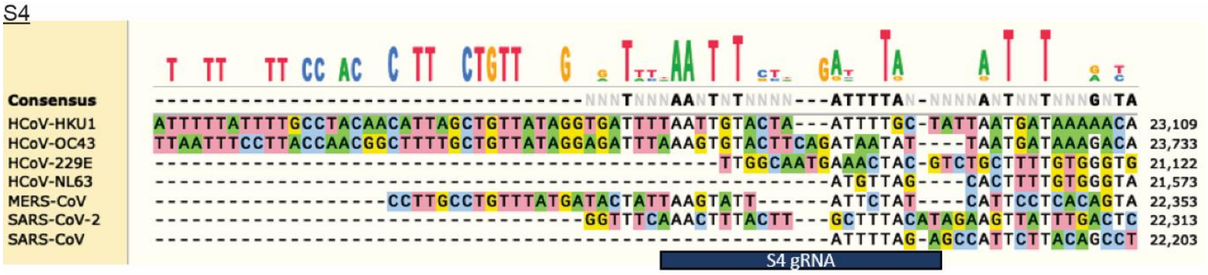
Consensus
HCoV-HKU1 23,109
HCoV-OC43 23,733
HCoV-229E 21,122
HCoV-NL63 21,573
MERS-CoV 22,353
SARS-CoV-2 22,313
SARS-CoV 22,203
S4 gRNA

S5

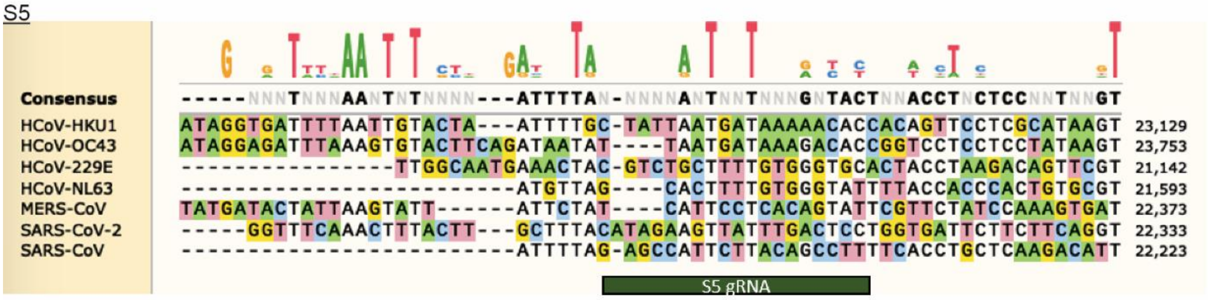
Consensus
HCoV-HKU1 23,129
HCoV-OC43 23,753
HCoV-229E 21,142
HCoV-NL63 21,593
MERS-CoV 22,373
SARS-CoV-2 22,333
SARS-CoV 22,223
S5 gRNA

S6

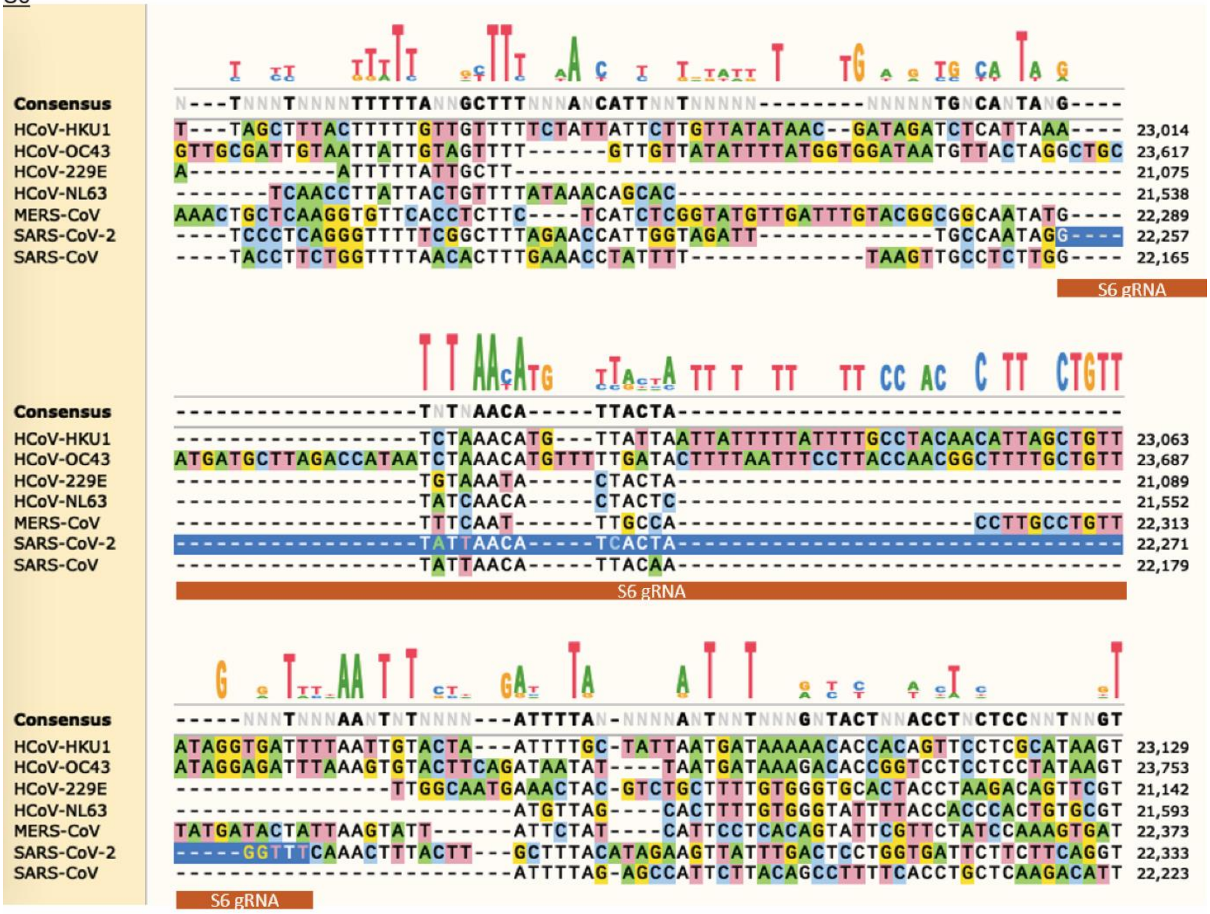
Consensus
HCoV-HKU1 23,014
HCoV-OC43 23,617
HCoV-229E 21,075
HCoV-NL63 21,538
MERS-CoV 22,289
SARS-CoV-2 22,257
SARS-CoV 22,165
S6 gRNA
Consensus
HCoV-HKU1 23,063
HCoV-OC43 23,687
HCoV-229E 21,089
HCoV-NL63 21,552
MERS-CoV 22,313
SARS-CoV-2 22,271
SARS-CoV 22,179
S6 gRNA
Consensus
HCoV-HKU1 23,129
HCoV-OC43 23,753
HCoV-229E 21,142
HCoV-NL63 21,593
MERS-CoV 22,373
SARS-CoV-2 22,333
SARS-CoV 22,223
S6 gRNA

S7

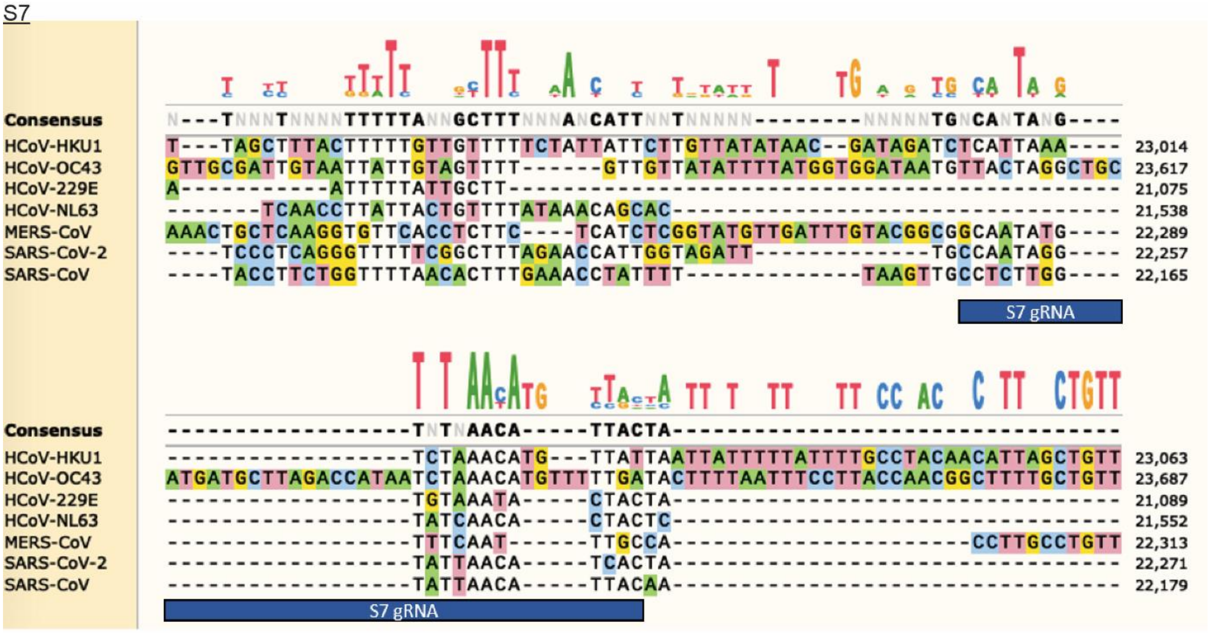
Consensus
HCoV-HKU1
HCoV-OC43
HCoV-229E
HCoV-NL63
MERS-CoV
SARS-CoV-2
SARS-CoV
23,014
23,617
21,075
21,538
22,289
22,257
22,165
S7 gRNA
23,063
23,687
21,089
21,552
22,313
22,271
22,179
S7 gRNA

S8, S9

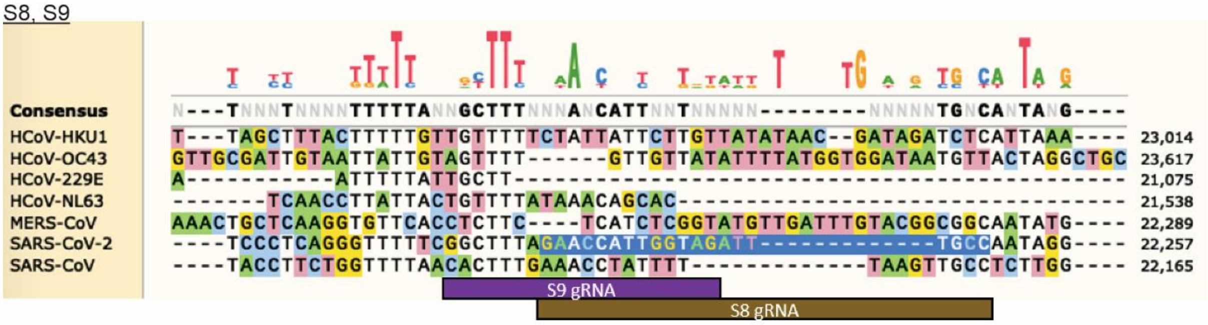
Consensus
HCoV-HKU1
HCoV-OC43
HCoV-229E
HCoV-NL63
MERS-CoV
SARS-CoV-2
SARS-CoV
23,014
23,617
21,075
21,538
22,289
22,257
22,165
S9 gRNA
S8 gRNA

S10

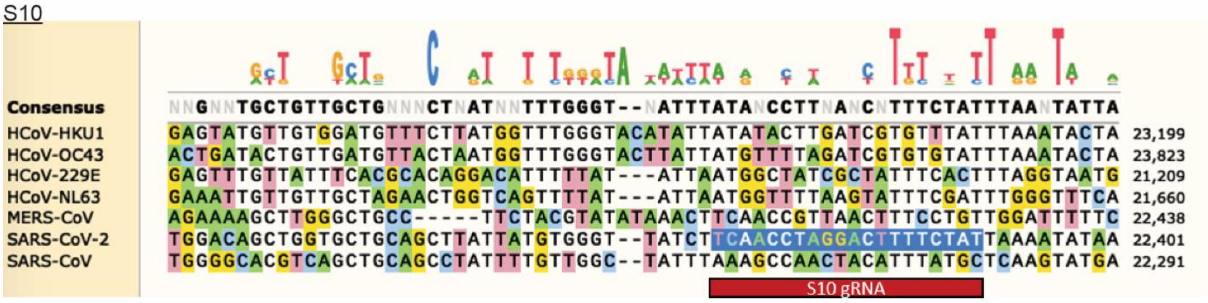
Consensus
HCoV-HKU1
HCoV-OC43
HCoV-229E
HCoV-NL63
MERS-CoV
SARS-CoV-2
SARS-CoV
23,199
23,823
21,209
21,660
22,438
22,401
22,291
S10 gRNA

S11, S12, S13, S14

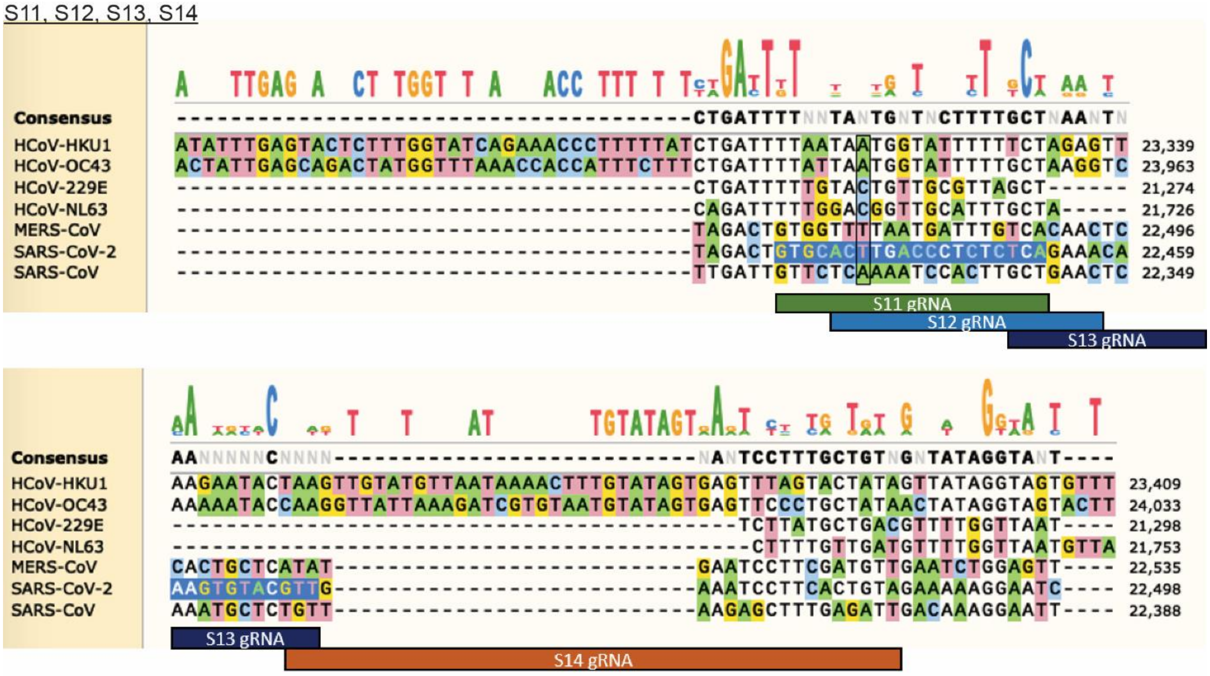

S15

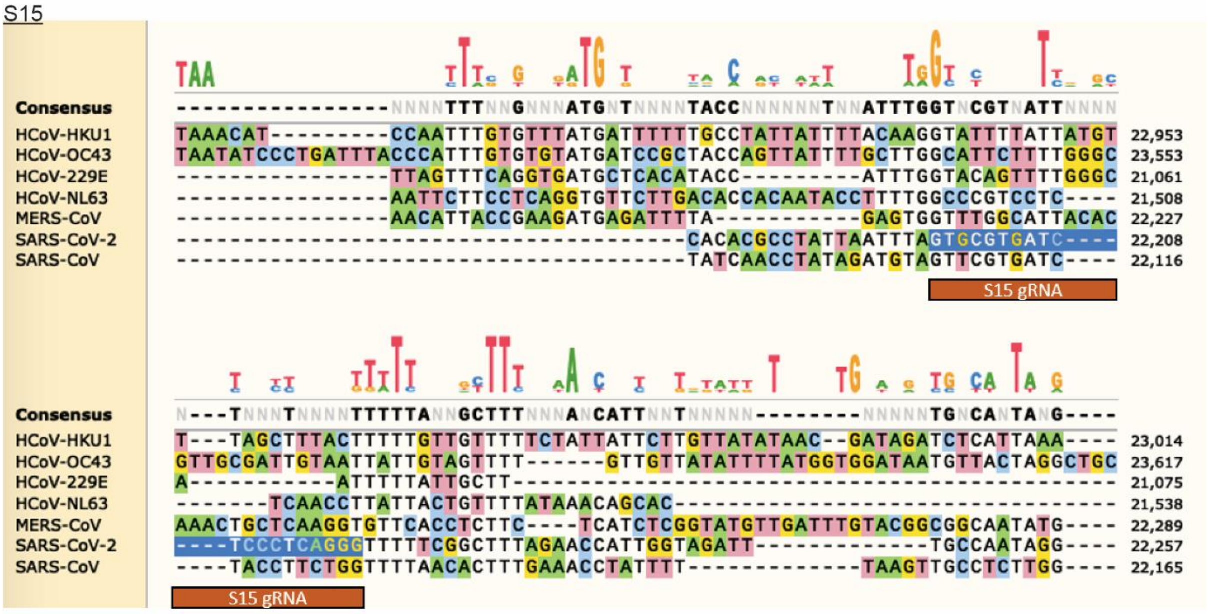

Supplementary Fig. 9 Multiple sequence alignment between seven coronaviruses, including SARS-CoV-2, showing the region of the S-gene targeted by the S4-S15 gRNAs that we evaluated with enAsCas12a in our study. The TTTV PAM precedes each 20nt spacer.

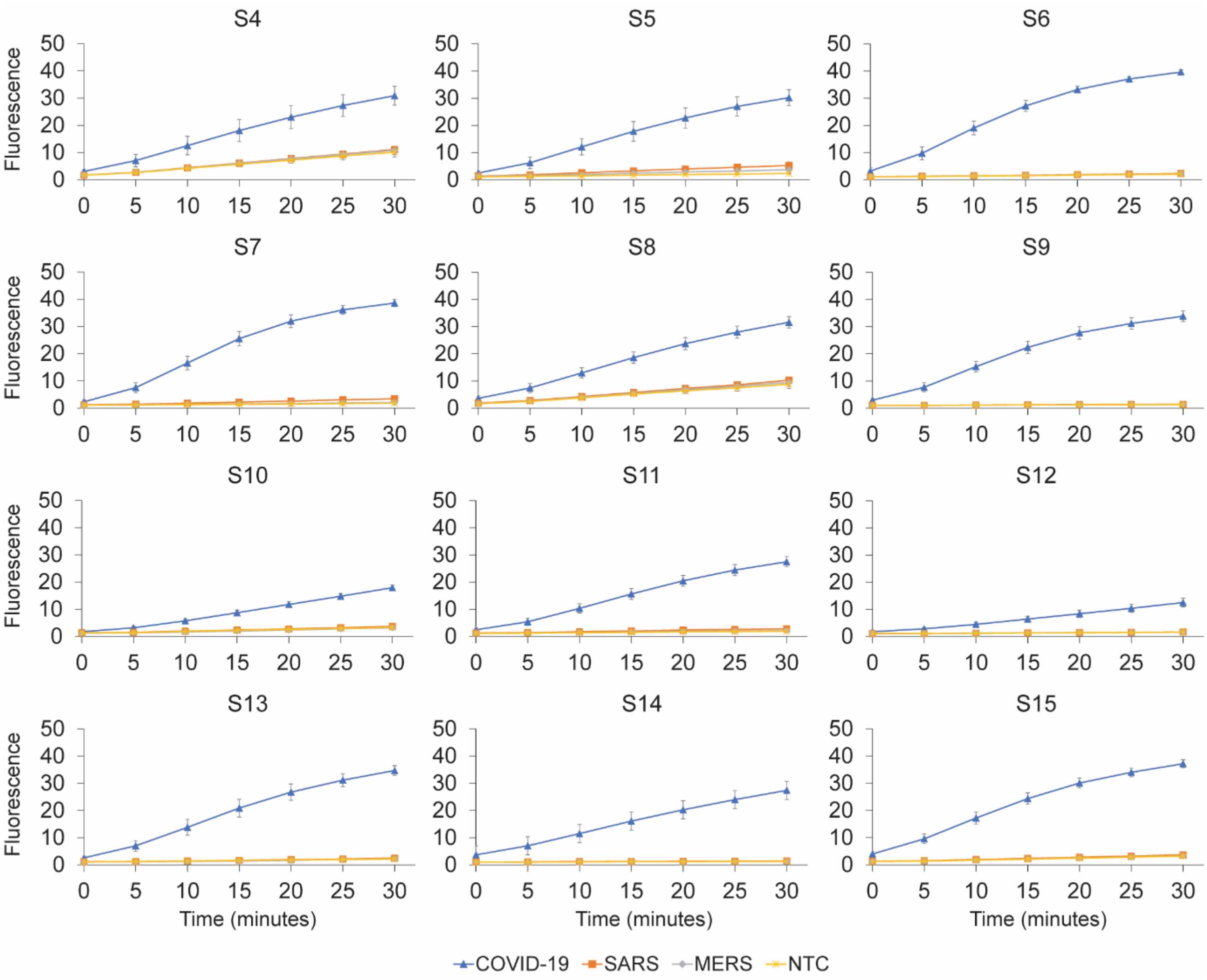

Supplementary Fig. 10 Time courses of the fluorescence intensity in our trans-cleavage assays for enAsCas12a complexed with various gRNAs of spacer length 20nt. The gRNAs were designed to target the S-gene of SARS-CoV-2. The assays were performed at 37°C and 1E11 copies of DNA were used as input. Data represent mean ± s.e.m. (n = 4 [S9], 5 [S4, S5, S6, S7, S11, S14], 6 [S8, S10, S12, S13], or 7 [S15] biological replicates).

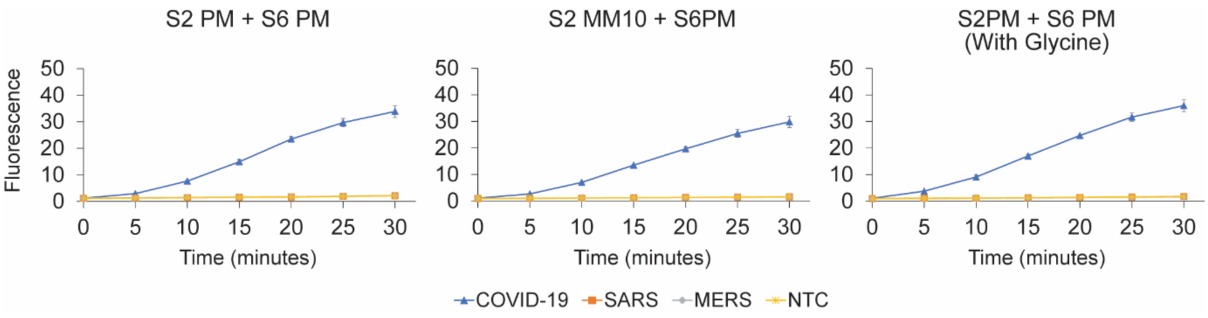

Supplementary Fig. 11 Time courses of the fluorescence intensity in our trans-cleavage assays for enAsCas12a complexed with two different gRNAs, each of which had a spacer length of 20nt. Left and middle panels: no glycine was used. Right panel: 8mM glycine was added. The assays were performed at 37°C and 1E11 copies of purified DNA template were used as input. Data represent mean ± s.e.m. (n = 3 [with glycine] or 4 [no glycine] biological replicates).

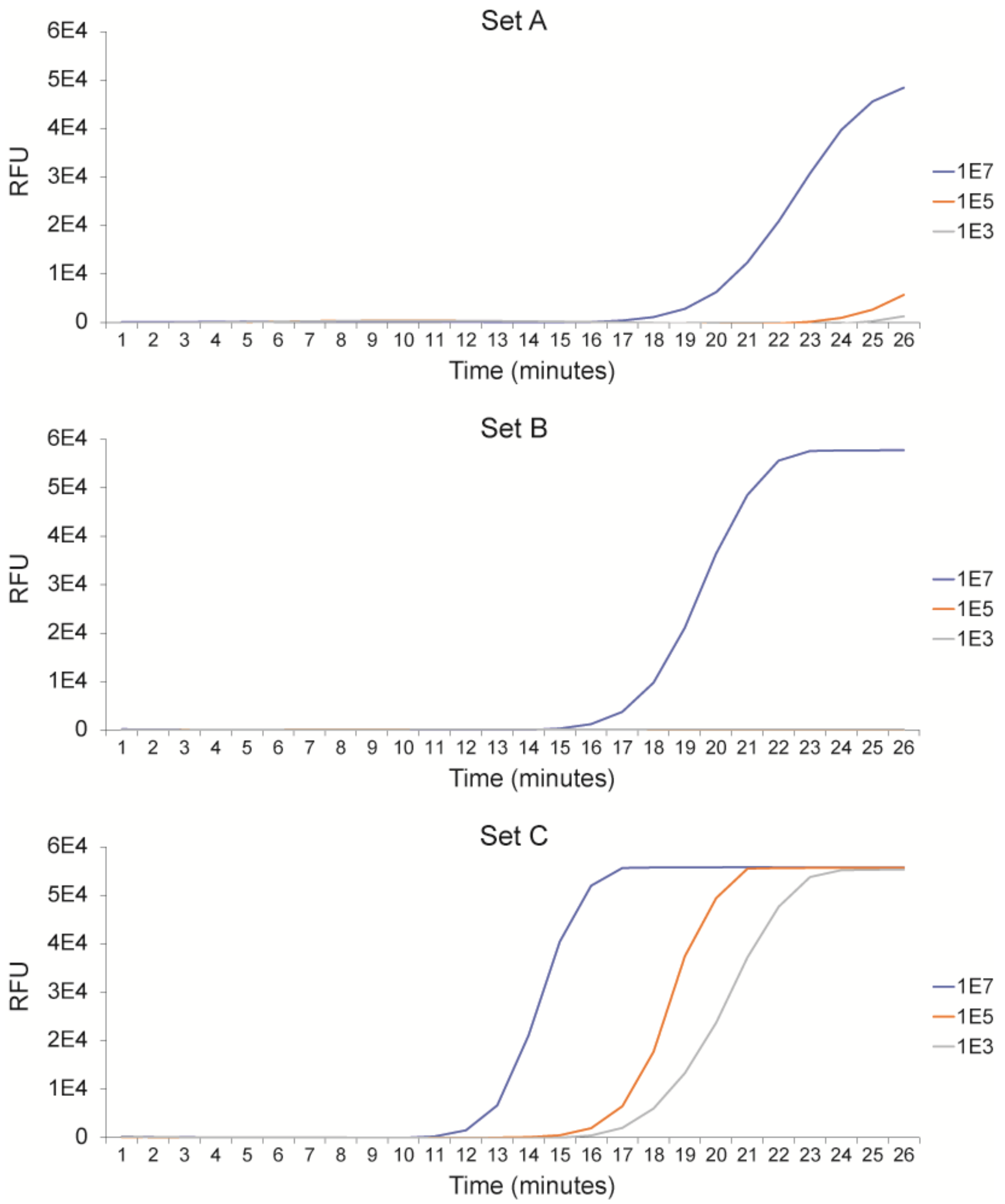

Supplementary Fig. 12 Real-time monitoring of the RT-LAMP reaction performed at 65°C for three different sets of primers targeting the S gene of SARS-CoV-2. Fluorescence signal was generated by the addition of a dye that was provided with the WarmStart LAMP kit (New England Biolabs). 1E3, 1E5, and 1E7 copies of synthetic SARS-CoV-2 RNA input were tested.

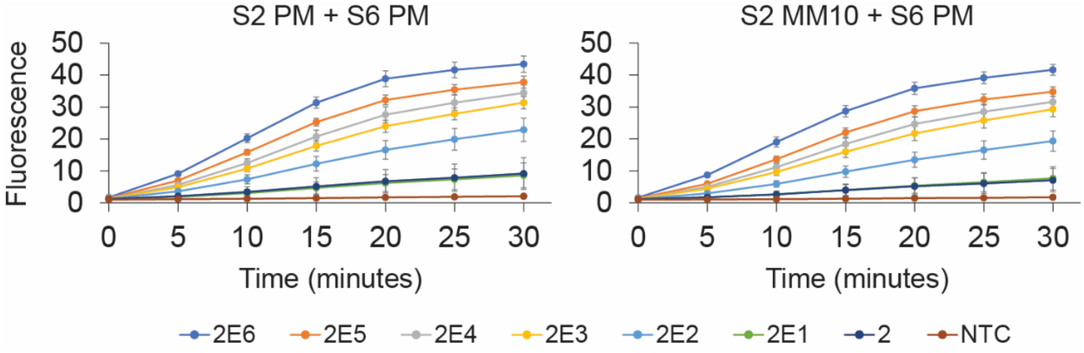

Supplementary Fig. 13 Time courses of the fluorescence intensity in our trans-cleavage assays for enAsCas12a complexed with both the S6 gRNA and either the perfect matched (PM) or a mismatched (MM10) S2 gRNA. Before the Cas detection reaction, various copies of synthetic SARS-CoV-2 RNA fragments (see legend) were used as input to an RT-LAMP reaction, which was performed at 65°C for 15 minutes with our initial set of LAMP primers (i.e. 0.2μM of each displacement primer, 1.6μM of each internal primer, and 0.8μM of each loop primer). Subsequently, 4μl LAMP products (out of 25μl) were used for the trans-cleavage assays, which were performed at 37°C. Fluorescence measurements were taken using a microplate reader at five-minute intervals. Data represent mean ± s.e.m. (n = 6 [2E6] or 7 [other copy numbers] biological replicates).

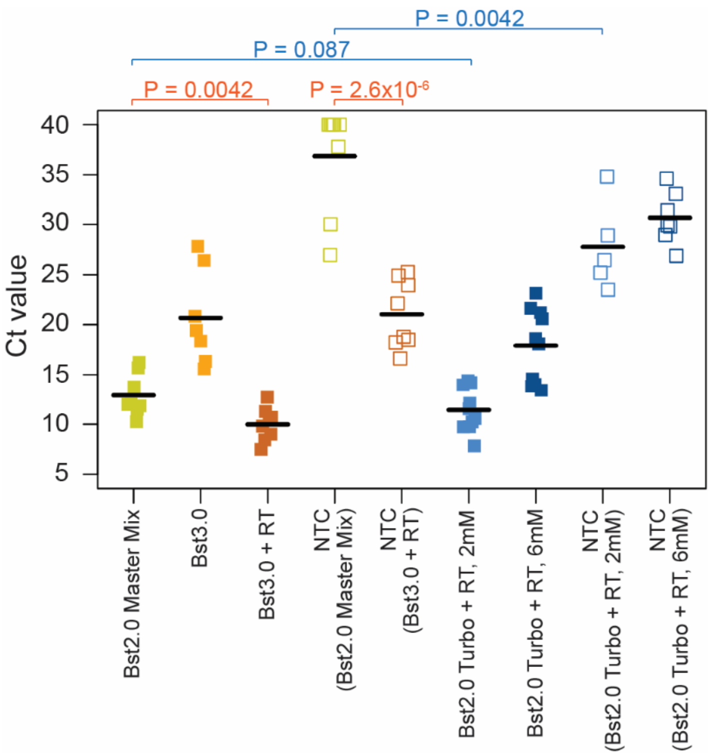

Supplementary Fig. 14 Strip chart showing the performance of various Bst DNA polymerases in RT-LAMP reactions. Here, every reaction contained the displacement primers (0.2µM each), the internal primers (1.6µM each), the loop primers (0.8µM each), and the swarm primers (1.6µM each). For most of our work, we have relied on the Bst2.0 mastermix, which contains a WarmStart reverse transcriptase to convert the RNA template into cDNA for the Bst2.0 enzyme to act on. Bst3.0 is an engineered DNA polymerase with enhanced reverse transcriptase activity, such that it is supposed to be capable of acting directly on RNA templates. In addition, the Saphir Bst2.0 Turbo Polymerase is supposed to give faster amplification rates as it contains an extra DNA-binding domain. The RT-LAMP reaction was carried out at 65°C in a real-time instrument with 20,000 copies of synthetic RNA template. The black horizontal bars among the data points in the strip chart represent the mean (n = 5 [NTC for 2mM Turbo], 7 [Bst3.0, NTC for 6mM Turbo], 8 [Master Mix, Bst3.0 + RT, NTC for Master Mix, NTC for Bst3.0 + RT], or 10 [2mM Turbo, 6mM Turbo] biological replicates). P values were calculated using one-sided Student's t-test.

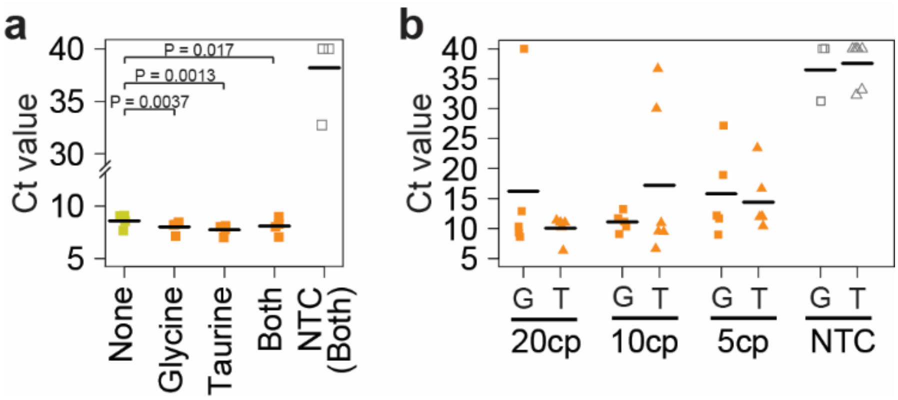

Supplementary Fig. 15 Effect of glycine and taurine on RT-LAMP.

a Strip chart showing how the kinetics of the LAMP module was altered by the addition of either 0.1M glycine or 50mM taurine. The RT-LAMP reaction was carried out at 65°C in a real-time instrument with 20,000 copies of SARS-CoV-2 synthetic RNA template. The black horizontal bars among the data points in the strip chart represent the mean (n = 4 biological replicates). P values were calculated using one-sided Student's t-test.

b Strip chart showing the sensitivity of the LAMP module in the presence of 0.1M glycine (G) or 50mM taurine (T). The RT-LAMP reaction was carried out at 65°C in a real-time instrument with variable copies of SARS-CoV-2 RNA template. The black horizontal bars among the data points in the strip chart represent the mean (n = 5 [glycine] or 6 [taurine] biological replicates).

F3	0.2	0.2	0.2	0.2	0.2	0.2	0.2	0.2
B3	0.2	0.4	0.2	0.2	0.4	0.2	0.4	0.4
FIP (PM)	0.8	0.8	1.6	0.8	0.8	1.6	0.8	0.8
FIP (tPM-3)	0.8	0.8	-	0.8	0.8	-	0.8	0.8
BIP (PM)	0.8	0.8	1.6	0.8	0.8	1.6	0.8	0.8
BIP (tPM-3)	0.8	0.8	-	0.8	0.8	-	0.8	0.8
LF	0.8	0.8	0.8	0.8	0.8	0.8	0.8	0.8
LB	0.8	0.8	0.8	0.8	0.8	0.8	0.8	0.8

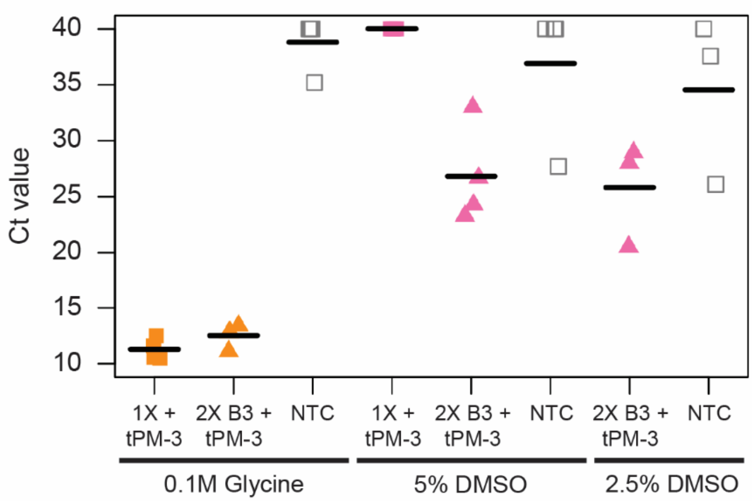

Supplementary Fig. 16 Strip chart showing how the sensitivity of the LAMP module was altered by the addition of either 5% or 2.5% DMSO. The RT-LAMP reaction was carried out at 65°C in a real-time instrument with 20 copies of RNA template corresponding to the S-gene of SARS-CoV-2. The black horizontal bars among the data points in the strip chart represent the mean (n = 3 [2.5% DMSO] or 4 [0.1M glycine, 5% DMSO] biological replicates).

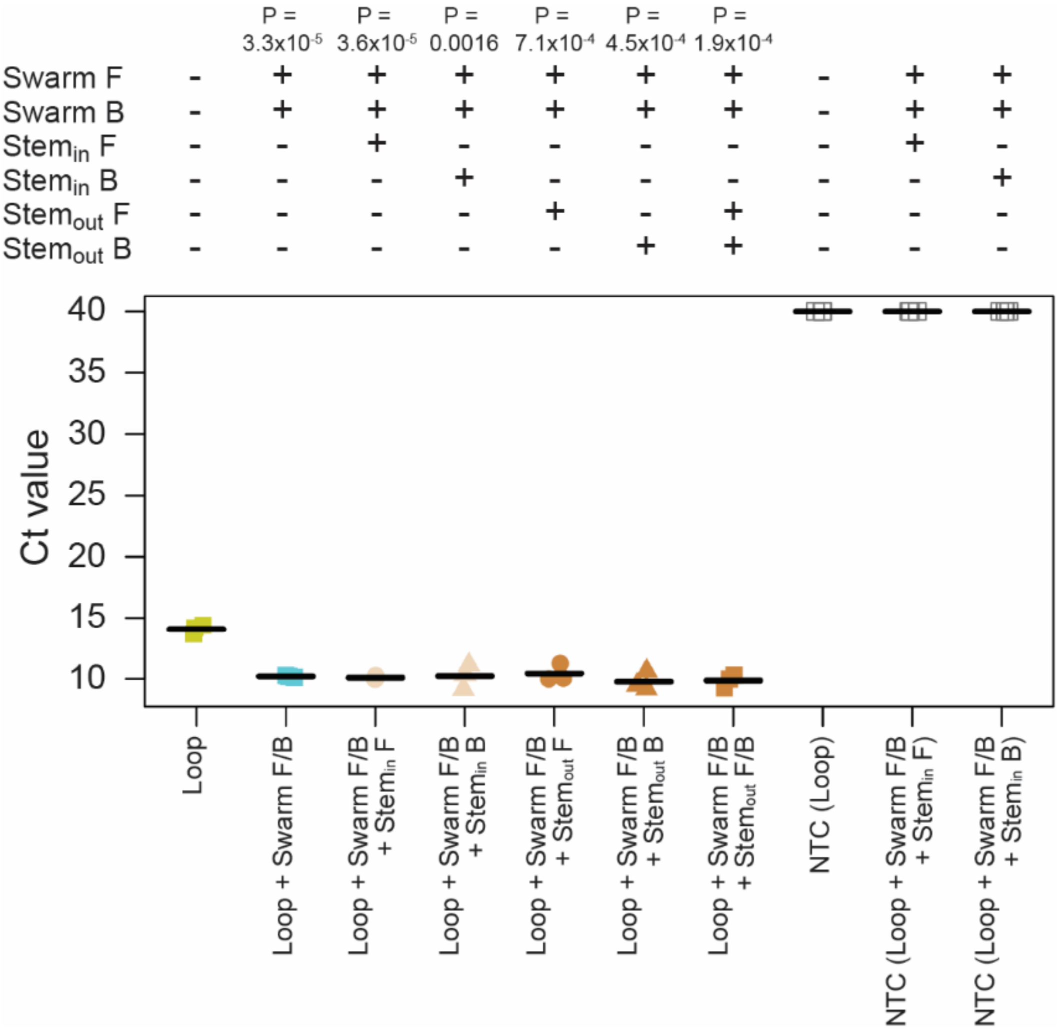

Supplementary Fig. 17 Strip chart showing the impact of swarm and stem primers on the kinetics of the LAMP reaction. Here, every reaction contained the displacement primers (0.2µM each), the internal primers (1.6µM each), and the loop primers (0.8µM each). Furthermore, it could also contain either two additional swarm primers or one or two additional stem primers (with the concentration of each extra primer being 1.6µM). The RT-LAMP reaction was carried out at 65°C in a real-time instrument with 20,000 copies of RNA template corresponding to the S-gene of SARS-CoV-2. The black horizontal bars among the data points in the strip chart represent the mean (n = 3 biological replicates). P values were calculated using one-sided Student's t-test.

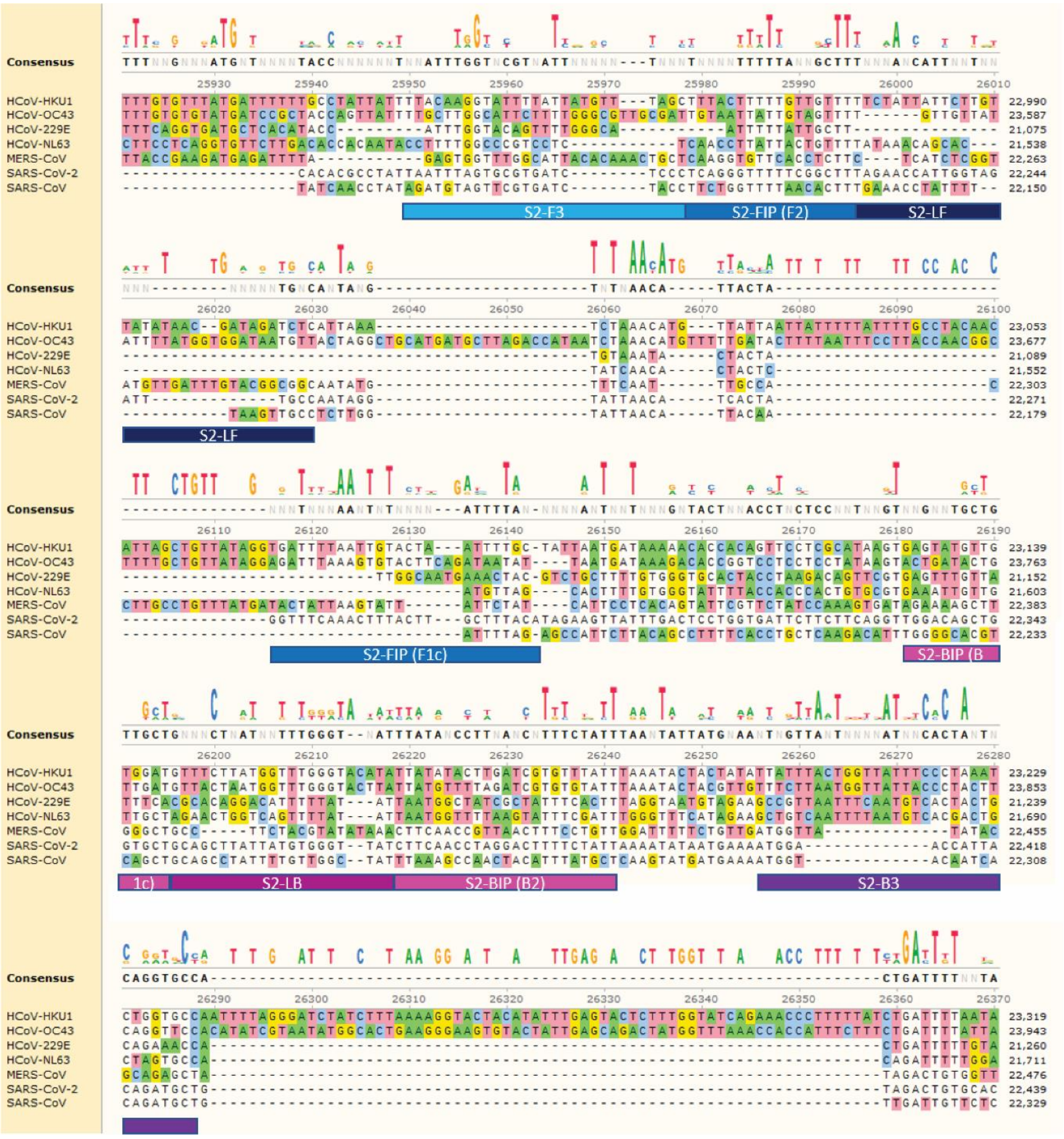

Supplementary Fig. 18 Multiple sequence alignment between seven coronaviruses, including SARS-CoV-2, showing the region of the S-gene targeted by our set of LAMP primers.

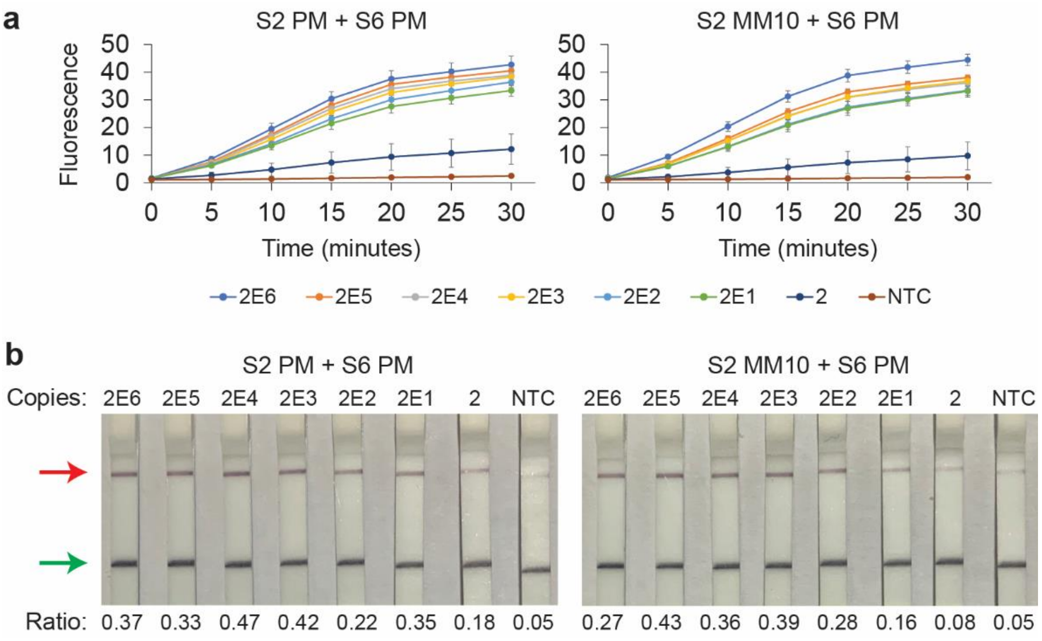

Supplementary Fig. 19 Mismatch tolerance by a two-gRNA system.

a Time courses of the fluorescence intensity in our trans-cleavage assays for enAsCas12a complexed with both the S6 gRNA and either the perfect matched (PM) or a mismatched (MM10) S2 gRNA. Before the Cas detection step, various copies of synthetic SARS-CoV-2 RNA fragments (see legend) were used as input to RT-LAMP, which was performed at 65°C for 15 minutes. The LAMP reaction conditions were optimized and encompassed 0.2µM of F3, 0.4µM of B3, 1.6µM of each full-length and 1nt-truncated internal primer, 0.8µM of each loop primer, 1.6µM of each swarm primer, 0.15U of Q5 high-fidelity DNA polymerase, and 0.1M glycine. Subsequently, 4µl of LAMP products were used for the trans-cleavage assays, which were performed at 37°C. Fluorescence measurements were taken using a microplate reader at five-minute intervals. Data represent mean ± s.e.m. (n = 5 [2E6] or 6 [other copy numbers] biological replicates).

b Robust detection of a SARS-CoV-2 S-gene sequence using a lateral flow assay. The enAsCas12a enzyme was complexed with both the S6 gRNA and either the perfect matched (PM) or a mismatched (MM10) S2 gRNA. Different copies of synthetic RNA fragments were used as input to the RT-LAMP reaction, which was performed at 65°C for 15 minutes under optimized conditions. Next, the Cas detection reaction was carried out at 37°C for 10 minutes before a dipstick was added to each reaction tube. Bands appeared on the dipstick by 2 minutes. The red arrow indicates the test bands, while the green arrow indicates the control bands.

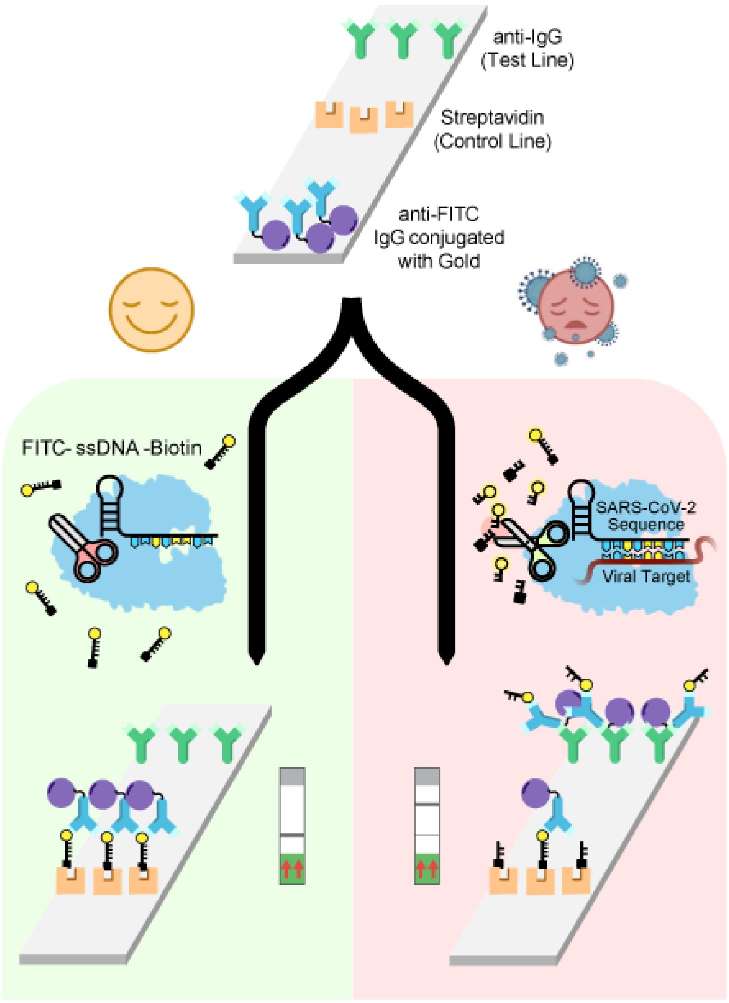

Supplementary Fig. 20 An illustration of the operating principle of a lateral flow strip or dipstick. On each strip are gold-conjugated IgG antibodies against the fluorophore near the sample pad, streptavidin immobilized at the control line, and antibodies against IgG immobilized at the test line. In the case of a virus-free sample, the Cas nuclease remains inactive and thus the reporter, comprising a fluorophore linked to biotin by a short piece of single-stranded DNA (ssDNA), stays intact. When the reaction is loaded on the strip, the gold-conjugated IgG first binds to the fluorophore and then the entire IgG-reporter complex is captured at the control line due to the high affinity of streptavidin for biotin. Consequently, a dark band is observed at the control line. However, in the case of an infected sample, the Cas nuclease cleaves its viral target, becomes hyperactivated, and then proceeds to cut the linker between the fluorophore and biotin. Subsequently, when the reaction is loaded on the strip, the gold-conjugated IgG still binds to the fluorophore, but the gold will not be deposited at the control line as the fluorophore is now free of biotin. Instead, the IgG-fluorophore complex continues flowing along the strip to the test line, where it is captured by the anti-IgG antibodies there. Consequently, a dark band is observed at the test line.

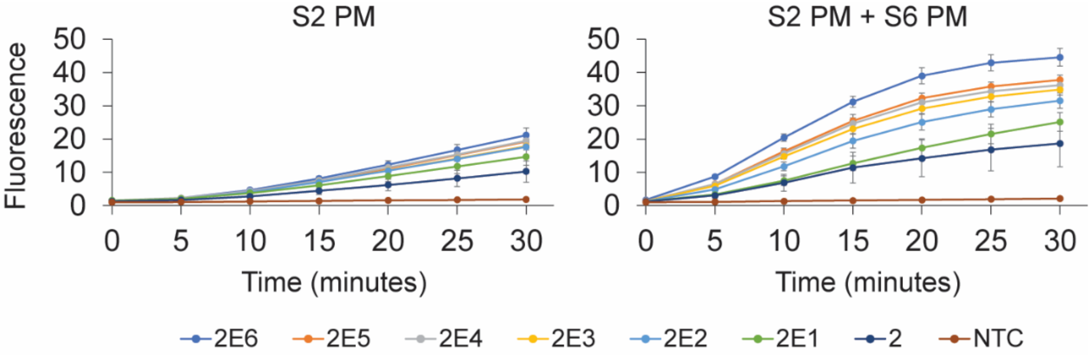

Supplementary Fig. 21 Time courses of the fluorescence intensity in our trans-cleavage assays for enAsCas12a assembled either with the S2 gRNA alone or with both the S2 and S6 gRNAs. These guides were designed to be perfect matched against the reference SARS-CoV-2 genome. Before the Cas detection reaction, various copies of *in vitro*-transcribed SARS-CoV-2 RNA fragments (see legend) bearing a known S254F mutation in the S-gene were used as input to an RT-LAMP reaction, which was performed at 65°C for 15 minutes under our optimized conditions. Subsequently, 4µl LAMP products (out of 25µl) were used for the trans-cleavage assays, which were performed at 37°C. Fluorescence measurements were taken at five-minute intervals using a microplate reader. Data represent mean ± s.e.m. (n = 5 biological replicates).

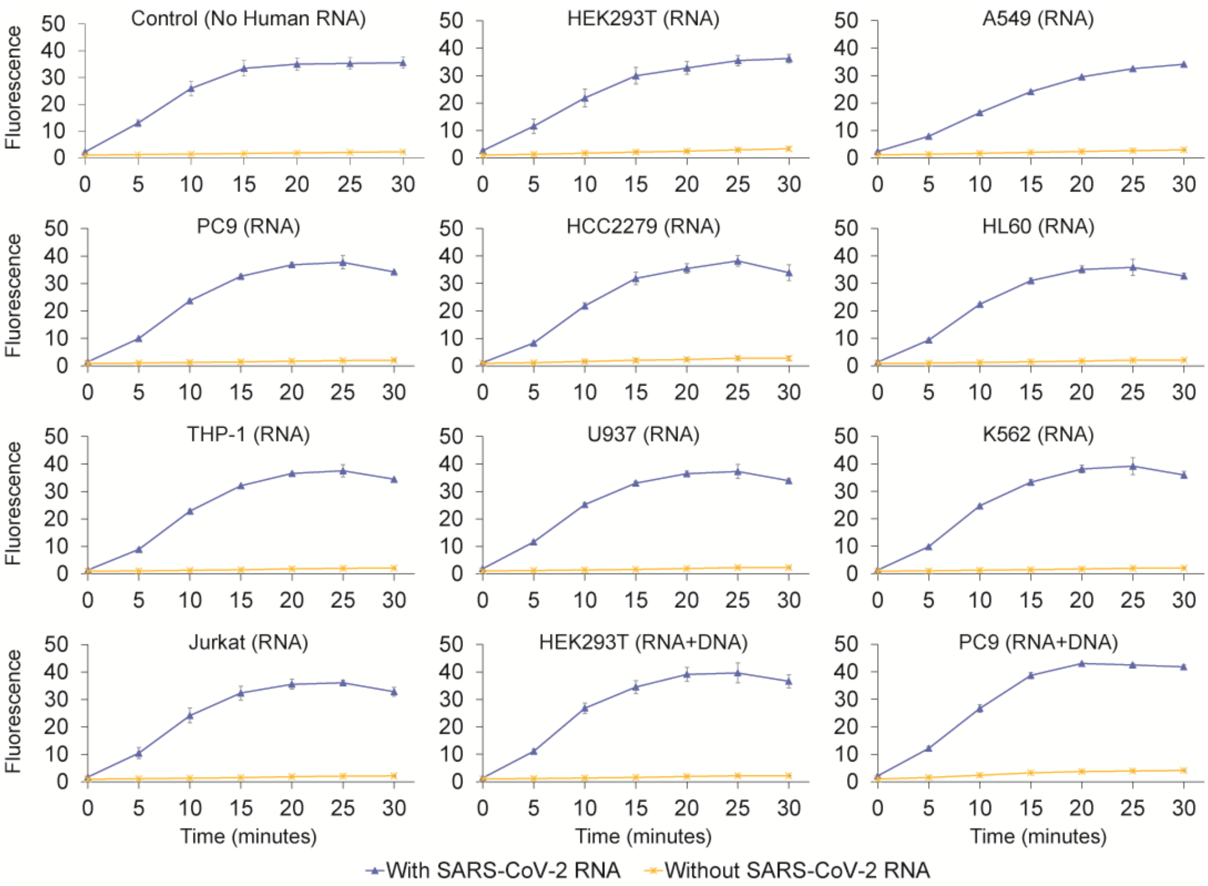

Supplementary Fig. 22 Time courses of the fluorescence intensity in our trans-cleavage assays for 20,000 copies of synthetic wildtype SARS-CoV-2 RNA fragments spiked into 10ng of total RNA from various immortalized human cell lines. Pure *in vitro*-transcribed viral RNA fragments (without any human RNA) were used as a control. The control or spiked RNA samples served as input to RT-LAMP, which was performed at 65°C for 15 minutes under our optimized conditions. Subsequently, 4μl LAMP products were used for the trans-cleavage assays, which were performed at 37°C. Fluorescence measurements were taken at five-minute intervals using a microplate reader. Data represent mean ± s.e.m. (n = 3 [all except control and HEK293T RNA], 6 [control], or 7 [HEK293T RNA] biological replicates).

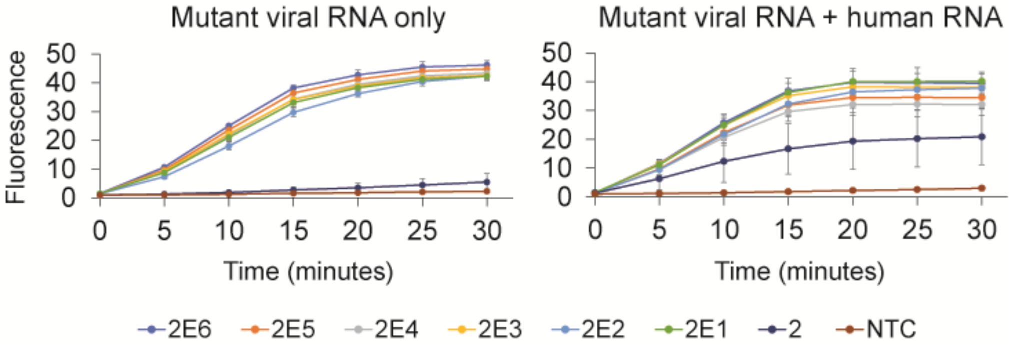

Supplementary Fig. 23 Time courses of the fluorescence intensity in our trans-cleavage assays for a S254F N234N double mutant RNA template either by itself or mixed with 10ng total human RNA from HCC2279 cells. A TCT-to-TTT mutation gave rise to the S254F amino acid change, while an AAC-to-AAT mutation was silent. Different copies of the *in vitro*-transcribed template (with or without human RNA) were used as input to the RT-LAMP reaction, which was performed at 65°C for 15 minutes under optimized conditions. 4μl LAMP products were then used for the trans-cleavage assays, which were performed at 37°C. Fluorescence measurements were taken at five-minute intervals using a microplate reader. Data represent mean ± s.e.m. (n = 4 biological replicates).

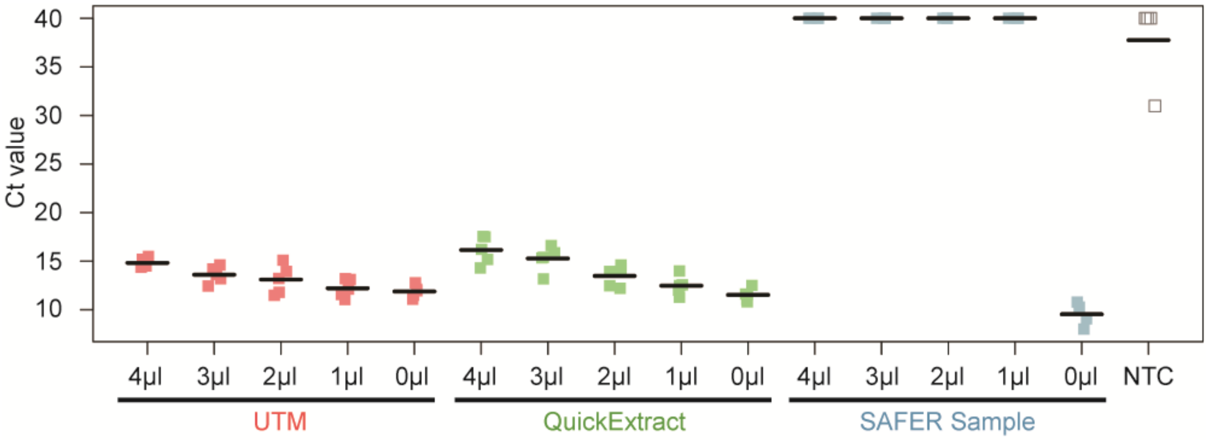

Supplementary Fig. 24 Strip chart showing the effect of various sample collection media on RT-LAMP. We tested 1-4µl of the widely used Universal Transport Medium (UTM), QuickExtract, and the SAFER Sample reagent. QuickExtract was heated at 95°C for 5 minutes before use to denature the Proteinase K in the solution. The RT-LAMP reaction was carried out at 65°C in a real-time instrument with 20,000 copies of RNA template. The black horizontal bars among the data points in the strip chart represent the mean (n = 4 [SAFER 0-4µl, NTC] or 5 [UTM 0-4µl, QuickExtract 0-4µl] biological replicates).

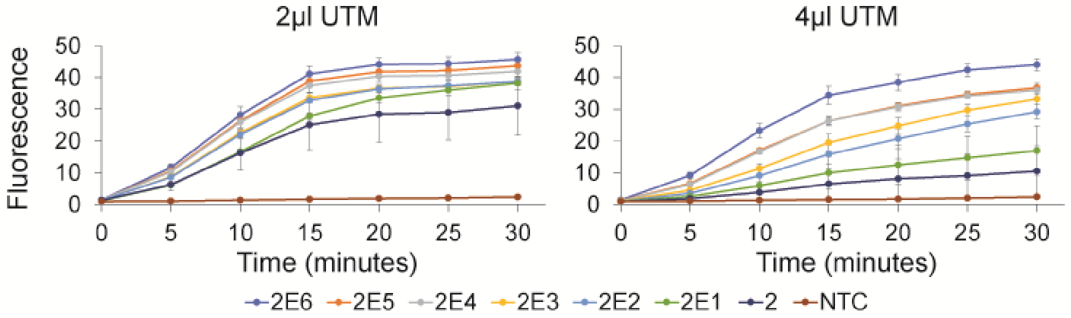

Supplementary Fig. 25 Time courses of the fluorescence intensity in our trans-cleavage assays for purified synthetic SARS-CoV-2 RNAs in the presence of 2μl or 4μl UTM. Various copies of *in vitro*-transcribed wildtype SARS-CoV-2 RNA fragments (see legend) in UTM were used as input to an RT-LAMP reaction, which was performed at 65°C for 15 minutes under our optimized conditions. Next, 4μl LAMP products (out of 25μl) were used for the trans-cleavage assays, which were performed at 37°C. Fluorescence measurements were taken at five-minute intervals using a microplate reader. Data represent mean ± s.e.m. (n = 4 biological replicates).

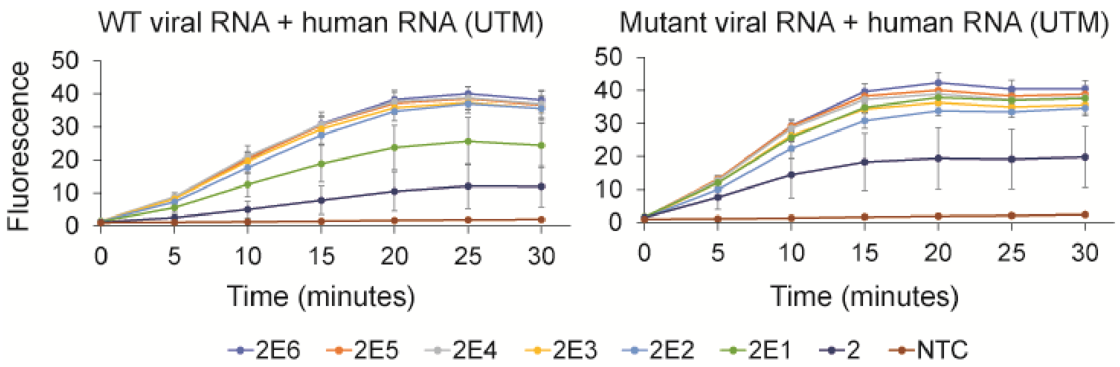

Supplementary Fig. 26 Time courses of the fluorescence intensity in our trans-cleavage assays for either wildtype or S254F N234N double mutant RNA template mixed with 10ng total human RNA from HCC2279 cells in the presence of 2µl UTM. Different copies of the *in vitro*-transcribed template (see legend) mixed with human RNA in UTM were used as input to the RT-LAMP reaction, which was performed at 65°C for 15 minutes under optimized conditions. 4µl LAMP products (out of 25µl) were then used for the trans-cleavage assays, which were performed at 37°C. Fluorescence measurements were taken at five-minute intervals using a microplate reader. Data represent mean ± s.e.m. (n = 4 biological replicates).

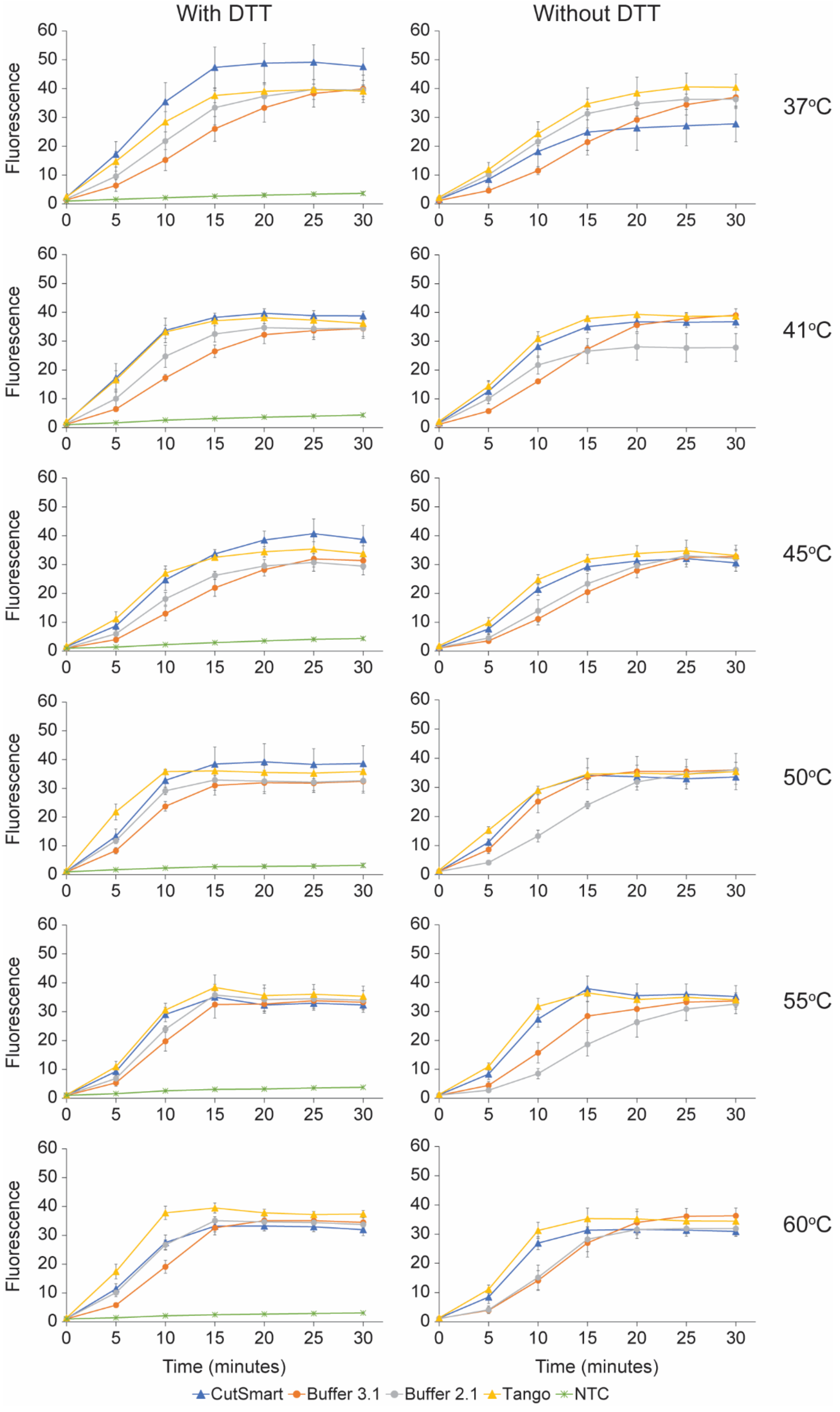

With DTT
Without DTT
37°C
41°C
45°C
50°C
55°C
60°C
Fluorescence
Time (minutes)
CutSmart
Buffer 3.1
Buffer 2.1
Tango
NTC

Supplementary Fig. 27 Time courses of the fluorescence intensity in our trans-cleavage assays, which were carried out at different temperatures. Various buffer conditions were evaluated as well. Here, enAsCas12a was complexed with the S2 gRNA only. 2E11 copies of synthetic DNA template corresponding to the S-gene of SARS-CoV-2 were used and the reaction volume was 50µl. Data represent mean ± s.e.m. (n = 3 [41°C, 45°C, 50°C, 55°C Tango alone, 60°C no DTT and NTC], 4 [37°C, 55°C all but Tango alone], or 6 [60°C with DTT] biological replicates).

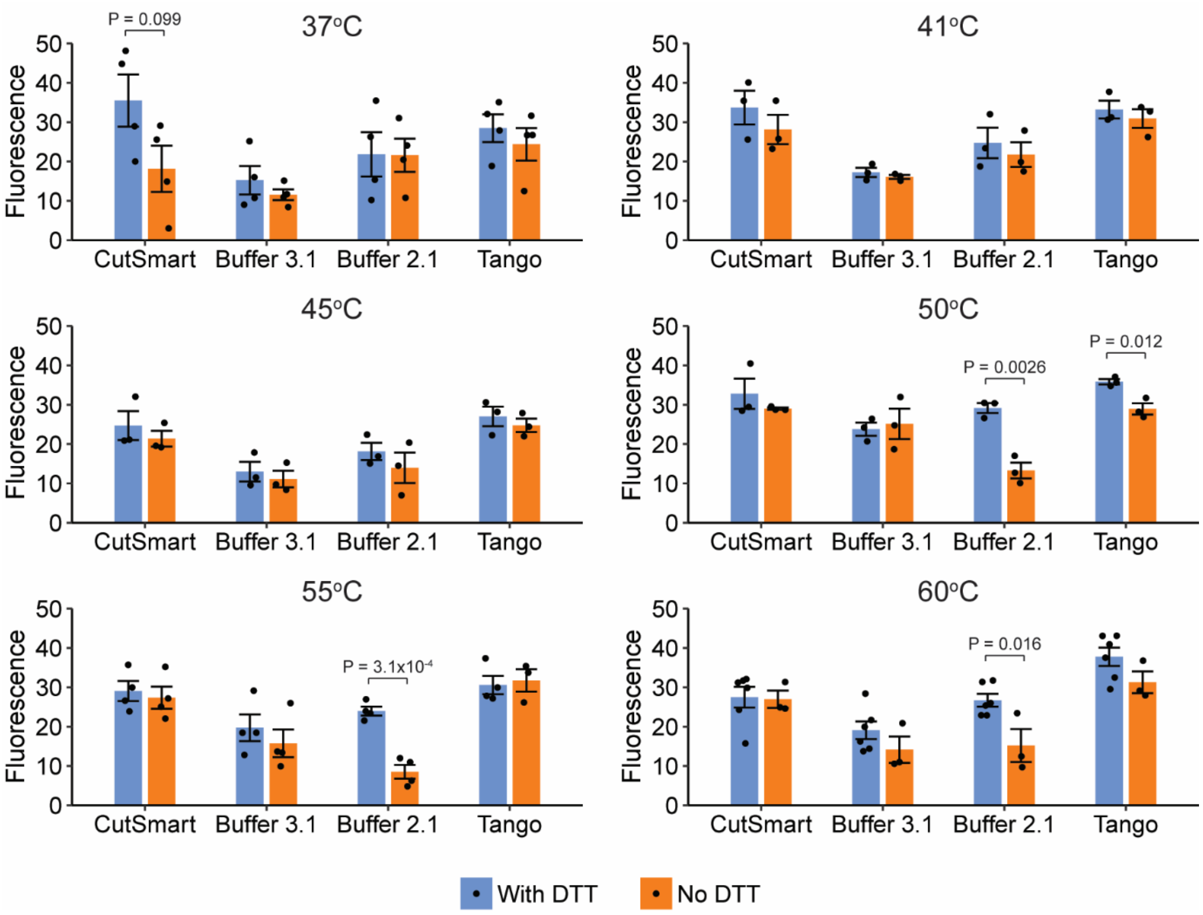

Supplementary Fig. 28 Effect of DTT on the collateral activity of enAsCas12a at different temperatures. 2E11 copies of synthetic DNA corresponding to the S-gene of SARS-CoV-2 were used in the experiment together with enAsCas12a complexed with the S2 gRNA only. Fluorescence measurements here were taken using a microplate reader after 10 minutes of cleavage reaction. DTT significantly improved the activity of enAsCas12a in CutSmart buffer at 37°C and in Buffer 2.1 at 50-60°C. Data represent mean ± s.e.m. (n = 3 [41°C, 45°C, 50°C, 55°C Tango alone, 60°C no DTT and NTC], 4 [37°C, 55°C all but Tango alone], or 6 [60°C with DTT] biological replicates). P values were calculated using two-sided Student's t-test.

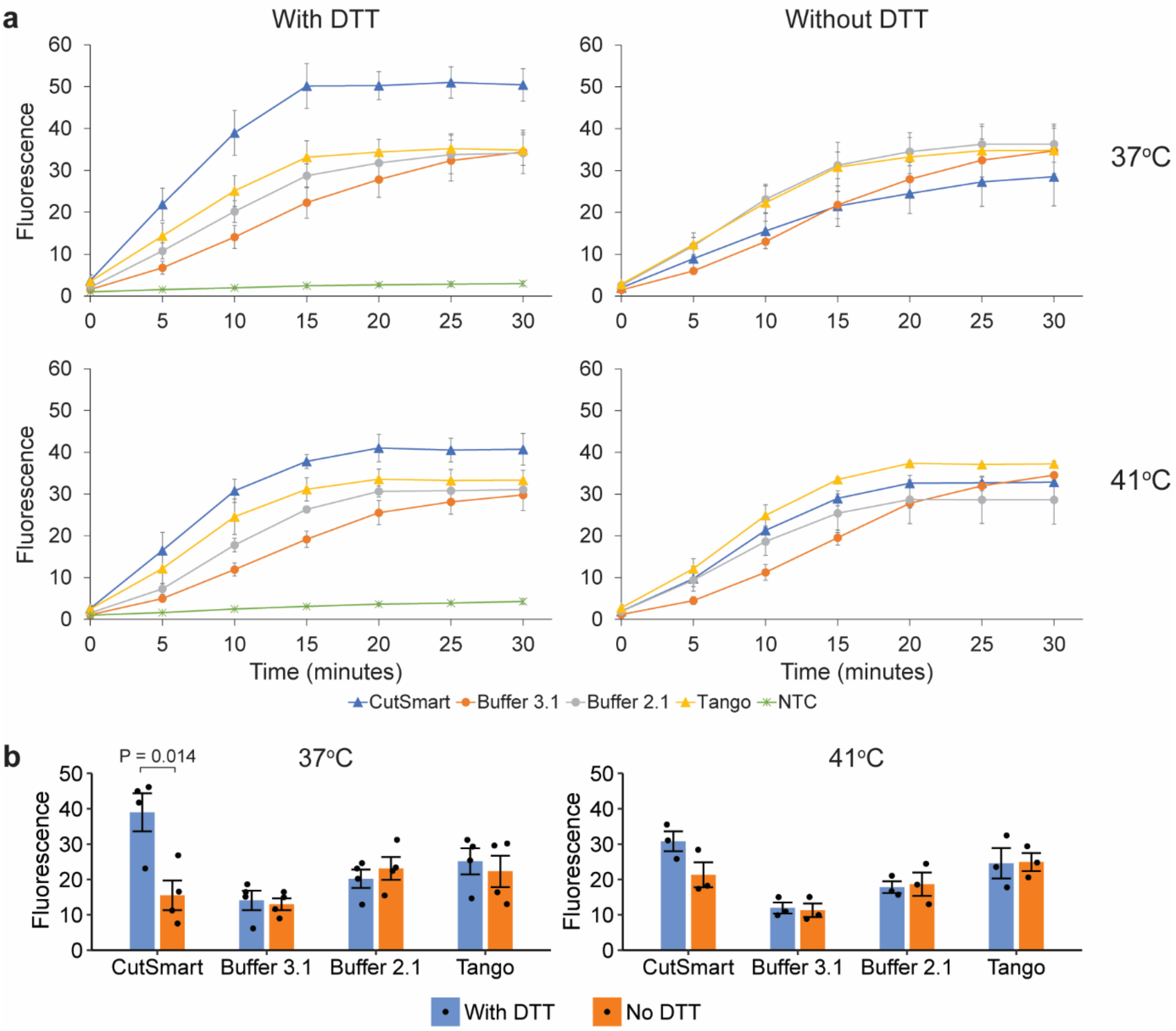

Supplementary Fig. 29 Collateral activity of enAsCas12a complexed with the S6-targeting gRNA only in different reaction buffers.

a Time courses of the fluorescence intensity in our trans-cleavage assays, which were carried out at either 37°C or 41°C in a 50µl reaction volume. 2E11 copies of synthetic DNA template corresponding to the S-gene of SARS-CoV-2 were used. Data represent mean ± s.e.m. (n = 3 [41°C] or 4 [37°C] biological replicates).

b Effect of DTT on the collateral activity of enAsCas12a with the S6 gRNA. Fluorescence measurements here were taken using a microplate reader after 10 minutes of cleavage reaction. DTT significantly improved the activity of enAsCas12a in CutSmart buffer at 37°C. Data represent mean ± s.e.m. (n = 3 [41°C] or 4 [37°C] biological replicates). P value was calculated using two-sided Student's t-test.

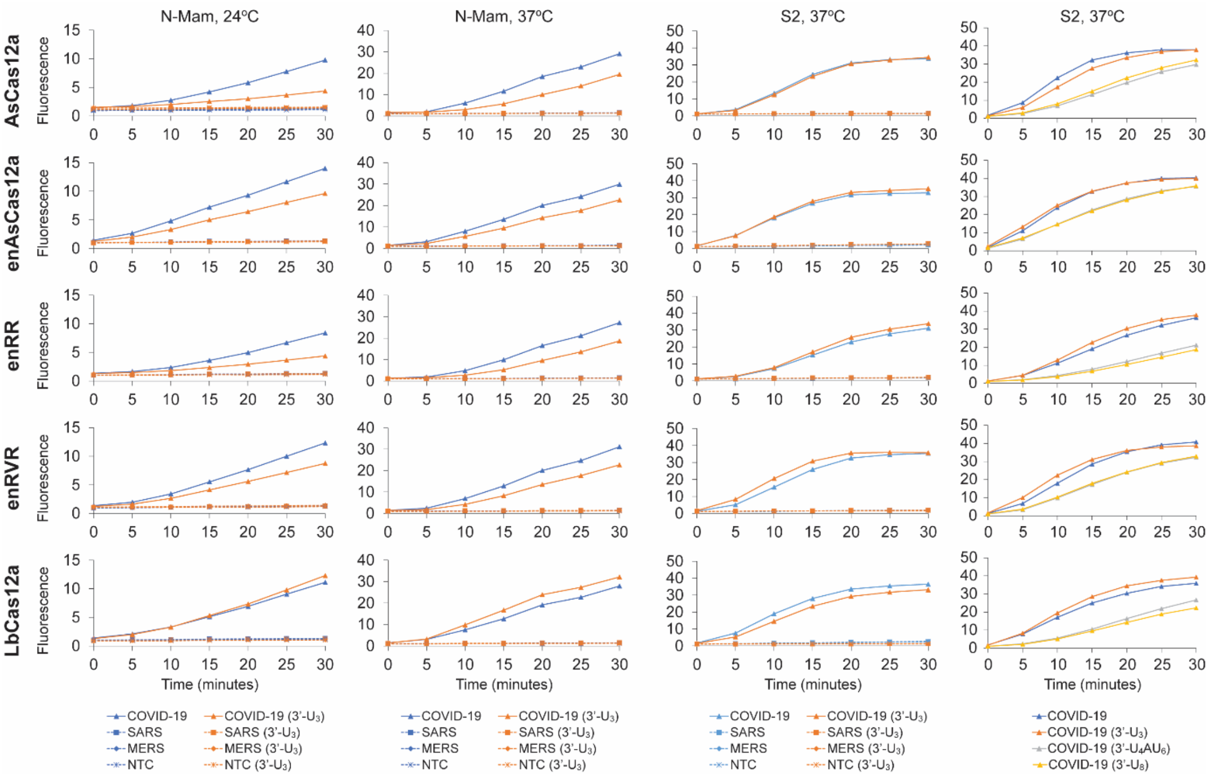

Supplementary Fig. 30 Effect of extending gRNAs at their 3' end on the collateral activity of Cas12a enzymes. 1E11 copies of DNA template were used as input to the trans-cleavage assays. Fluorescence measurements were taken at five-minute intervals using a microplate reader and all the measurements were normalized to the no-template control (NTC) at the 0min timepoint. We generated a new N-Mam gRNA with a U_3 3'-overhang and compared it with the original unmodified gRNA at 24°C and 37°C, but found that the 3'-extended gRNA yielded lower fluorescence signals for AsCas12a and its variants. We also generated new S2 gRNAs with a U_3, U_8, or U_4AU_6 3'-overhang and compared them with the original unmodified gRNA at 37°C. The gRNA with a U_3 3'-overhang gave similar fluorescence signals as the original gRNA, while the gRNA with a U_8 or U_4AU_6 3'-overhang yielded poorer fluorescence output for all the tested Cas12a enzymes.

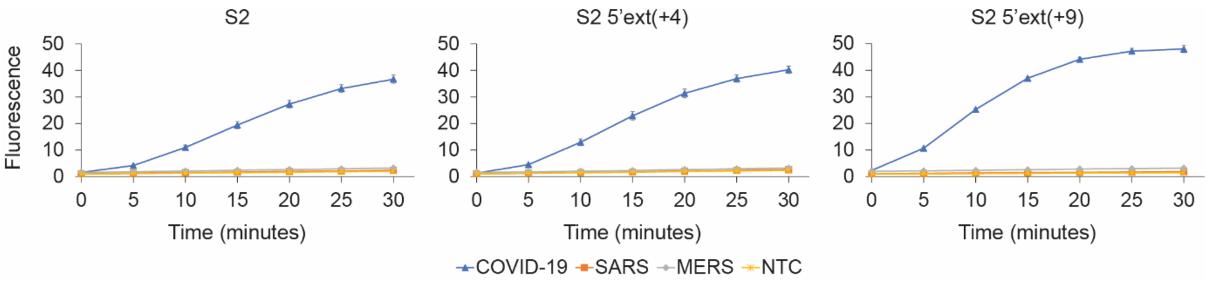

Supplementary Fig. 31 Comparison of 5'-extended gRNAs with the original S2-targeting gRNA. To monitor the enAsCas12a-mediated cleavage reaction at 37°C, fluorescence measurements were taken at five-minute intervals using a microplate reader. 2E11 copies of synthetic DNA template were used. All readings were normalized to NTC at the 0min timepoint. Data represent mean ± s.e.m. (n = 6 [S2 5'ext(+9)] or 11 [S2, S2 5'ext(+4)] biological replicates).

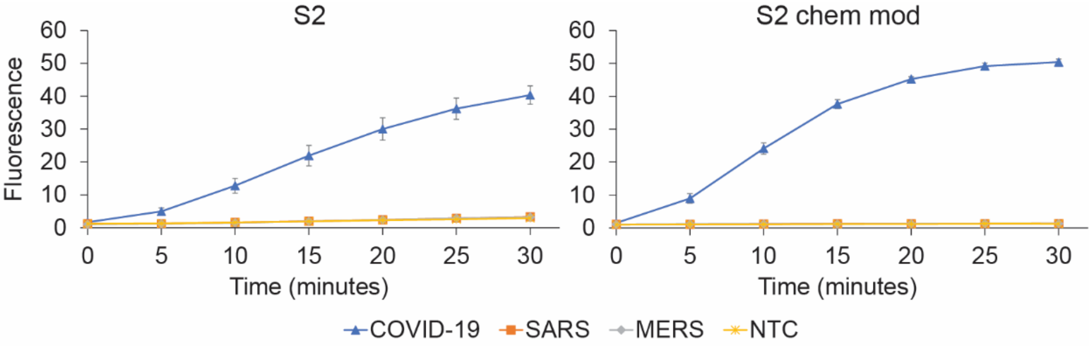

Supplementary Fig. 32 Comparison of a chemically modified gRNA containing 2'-O-methyl ribonucleotides, 2'-deoxy-2'-fluoro-ribonucleotides, and phosphorothioate bonds with the original S2-targeting gRNA. To monitor the enAsCas12a-mediated cleavage reaction at 37°C, fluorescence measurements were taken at five-minute intervals using a microplate reader. 2E11 copies of synthetic DNA template were used. All readings were normalized to NTC at the 0min timepoint. Data represent mean ± s.e.m. (n = 3 [S2 SARS, S2 MERS], 6 [S2 chem mod SARS, S2 chem mod MERS], or 8 [COVID-19, NTC] biological replicates).

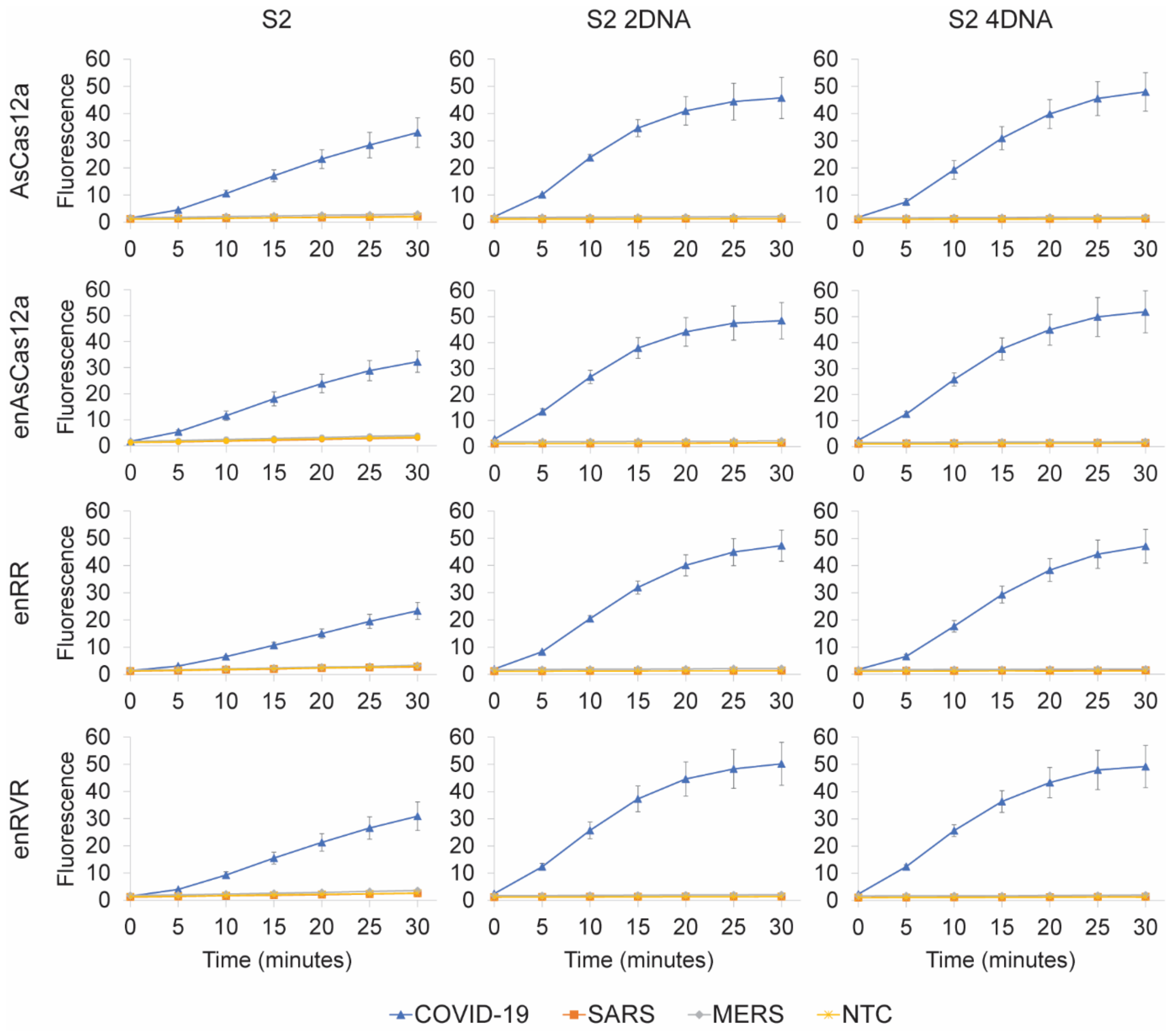

Supplementary Fig. 33 Comparison of DNA-RNA hybrid guides with the original unmodified S2-targeting gRNA. To monitor the cleavage reaction at 37°C, fluorescence measurements were taken at five-minute intervals using a microplate reader. 2E11 copies of synthetic DNA template were used. All readings were normalized to NTC at the 0min timepoint. Data represent mean ± s.e.m. (n = 3 biological replicates).

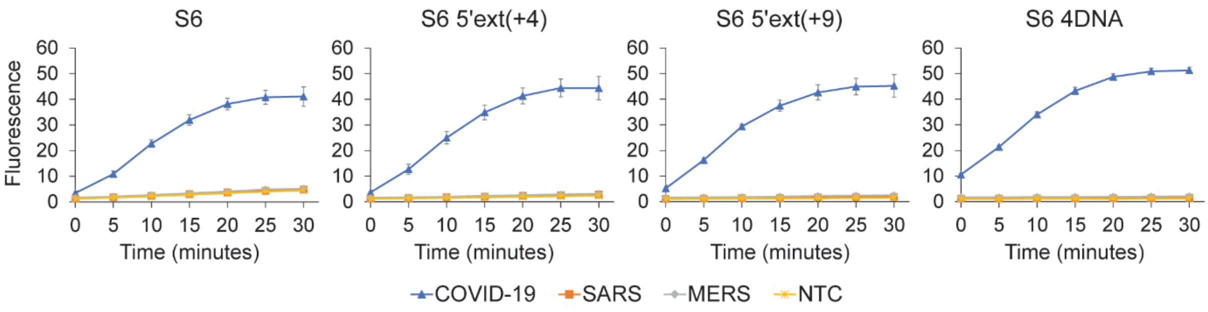

Supplementary Fig. 34 Comparison of 5’-extended gRNAs and a DNA-RNA hybrid guide with the original S6-targeting gRNA. To monitor the enAsCas12a-mediated cleavage reaction at 37°C, fluorescence measurements were taken at five-minute intervals using a microplate reader. 2E11 copies of synthetic DNA template were used. All readings were normalized to NTC at the start of the experiment. Data represent mean ± s.e.m. (n = 3 [S6 4DNA] or 4 [S6, S6 5'ext(+4), S6 5'ext(+9)] biological replicates).

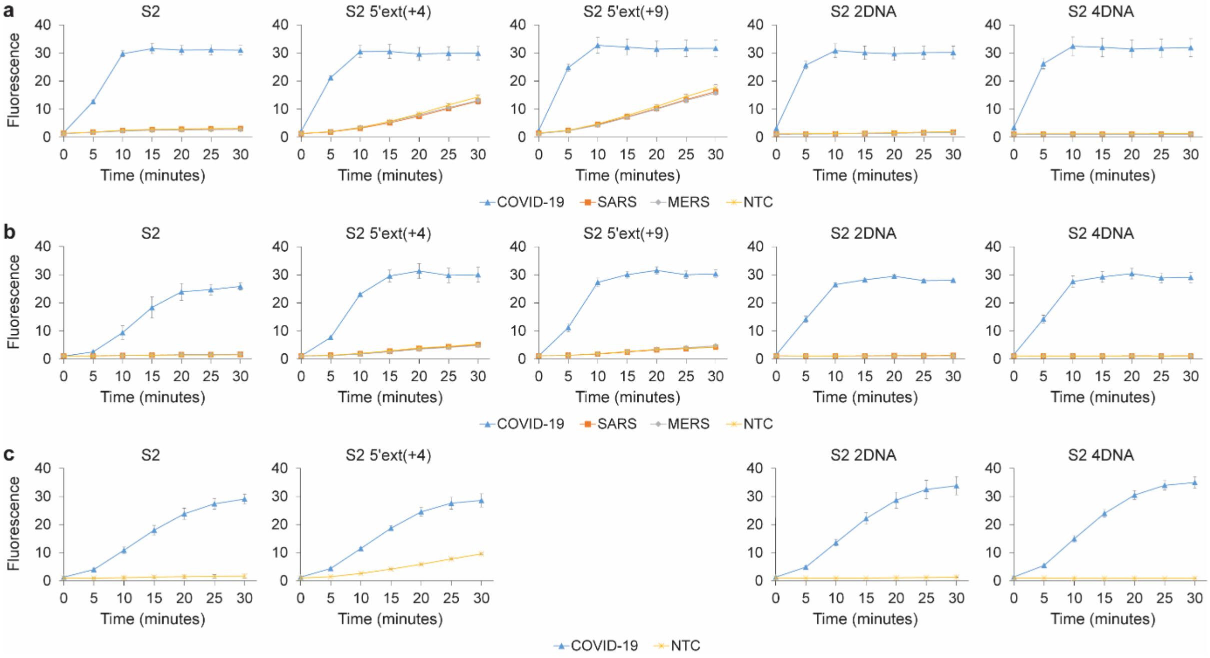

Supplementary Fig. 35 Comparison of 5’-extended gRNAs and DNA-RNA hybrid guides with the original S2-targeting gRNA. To monitor the enAsCas12a-mediated cleavage reaction at 60°C, fluorescence measurements were taken at five-minute intervals using a microplate reader. The trans-cleavage assays were performed in (**a**) Tango buffer, (**b**) Buffer 3.1 with DTT, or (**c**) CutSmart buffer with DTT. 2E11 copies of synthetic DNA template were used. All readings were normalized to NTC at the 0min timepoint. Data represent mean ± s.e.m. (n = 3 biological replicates).

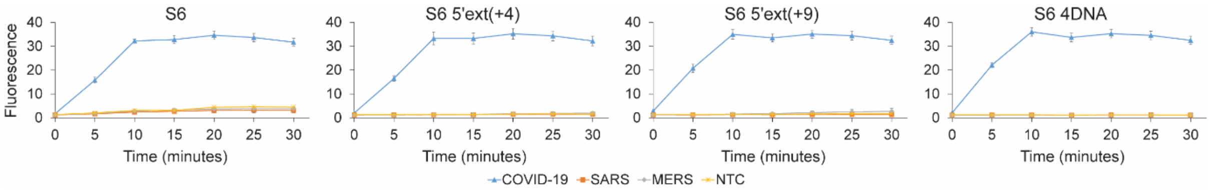

Supplementary Fig. 36 Comparison of 5’-extended gRNAs and a DNA-RNA hybrid guide with the original S6-targeting gRNA. To monitor the enAsCas12a-mediated cleavage reaction at 60°C, fluorescence measurements were taken at five-minute intervals using a microplate reader. The trans-cleavage assays were performed in Tango buffer. 2E11 copies of synthetic DNA template were used. All readings were normalized to NTC at the 0min timepoint. Data represent mean ± s.e.m. (n = 3 biological replicates).

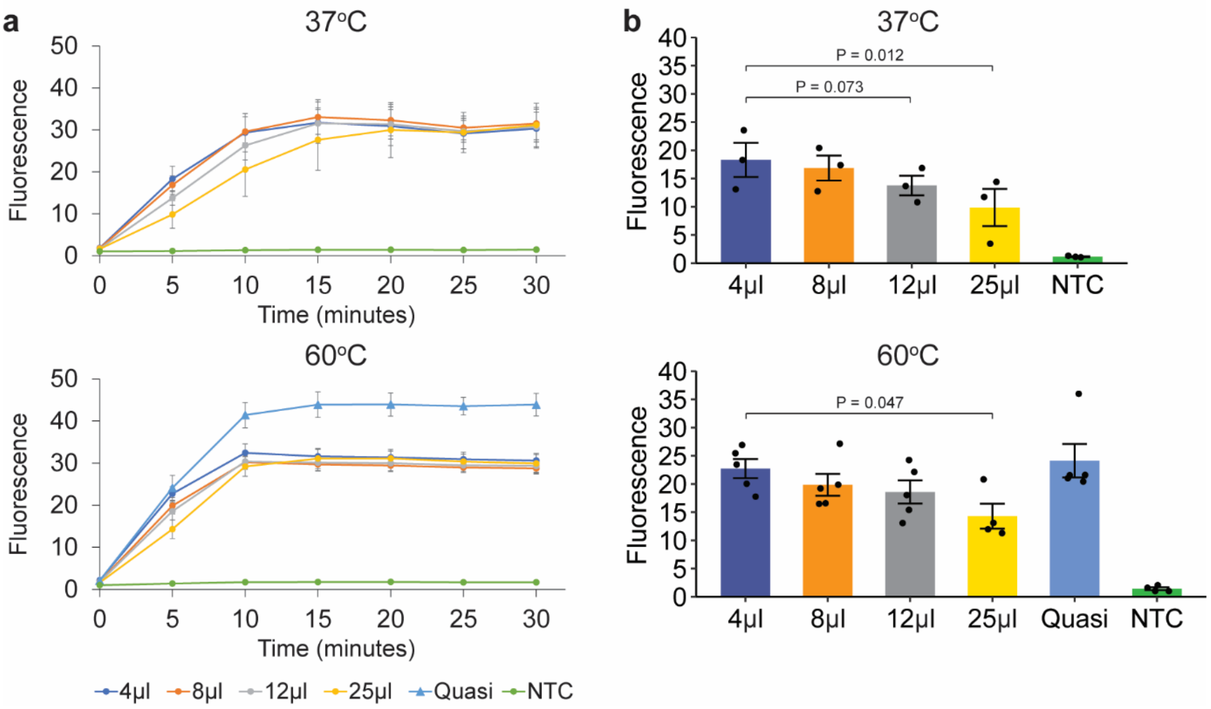

Supplementary Fig. 37 Effect of different volumes of LAMP products on the CRISPR module. **a** Time courses of the fluorescence intensity in our trans-cleavage assays for enAsCas12a complexed with both the S2 4DNA hybrid guide and the S6 9nt 5'-extended gRNA. Prior to the Cas detection step, RT-LAMP was performed at 65°C for 15 minutes with 20,000 copies of synthetic SARS-CoV-2 RNA template as input. Different amounts of LAMP products (4µl, 8µl, 12µl, or 25µl) were added into the CRISPR reaction mix before the cleavage assays were carried out at either 37°C or 60°C. CutSmart buffer with DTT was used. Fluorescence readings were taken at 5-minute intervals using a microplate reader and were then normalized to NTC at the 0min timepoint. Data represent mean ± s.e.m. (n = 3 [37°C], 4 [60°C 25µl and NTC], or 5 [60°C 4-12µl and quasi] biological replicates).

b Summary of enAsCas12a's collateral activity when different amounts of LAMP products were added into the CRISPR reaction mix. "Quasi" refers to the setup where the CRISPR mix was added into the LAMP reaction tube instead. The fluorescence measurements here were taken after 5 minutes of cleavage reaction. Data represent mean ± s.e.m. (n = 3 [37°C], 4 [60°C 25µl and NTC], or 5 [60°C 4-12µl and quasi] biological replicates). P values were calculated using two-sided Student's t-test.

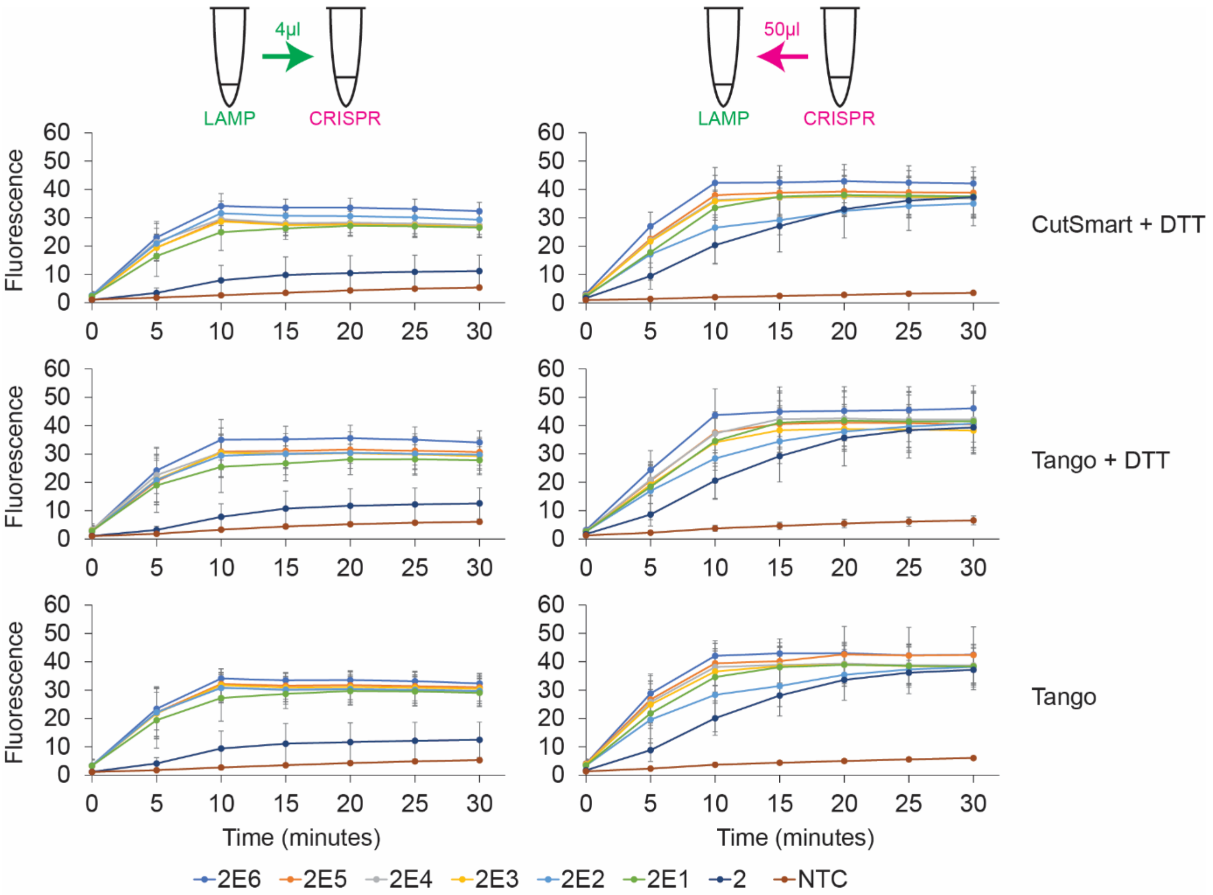

Supplementary Fig. 38 Analytical LoD of our assay based on either the original workflow (left) or when all LAMP products were utilized (right). Different copies of *in vitro* transcribed SARS-CoV-2 RNA (see legend) were used as input to the RT-LAMP reaction, which was performed at 65°C for 15 minutes. Subsequently, the Cas detection reaction was carried out at 60°C, with the fluorescence measurements taken at 5-minute intervals using a microplate reader. Data represent mean ± s.e.m. (n = 3 biological replicates).

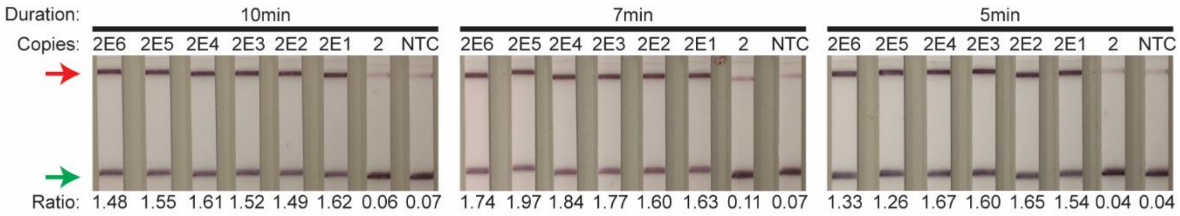

Supplementary Fig. 39 Analytical LoD of synthetic wildtype SARS-CoV-2 RNA by enAsCas12a complexed with both the S2 4DNA hybrid guide and the S6 9nt 5'-extended gRNA in the presence of 2µl UTM. Different copies of synthetic viral template in clean UTM were used as input to the RT-LAMP reaction, which was performed at 65°C for 15 minutes. Next, 50µl of CRISPR reaction mix in Tango buffer was added directly into each LAMP reaction tube. The trans-cleavage assay was then carried out at 60°C for 5, 7, or 10 minutes before a dipstick was inserted into each tube. Bands appeared on the dipsticks by 2 minutes. The red arrow indicates the test bands, while the green arrow indicates the control bands. Strong test bands were detected from 2E1 to 2E6 copies of viral RNA even with only 5 minutes of cleavage reaction time.

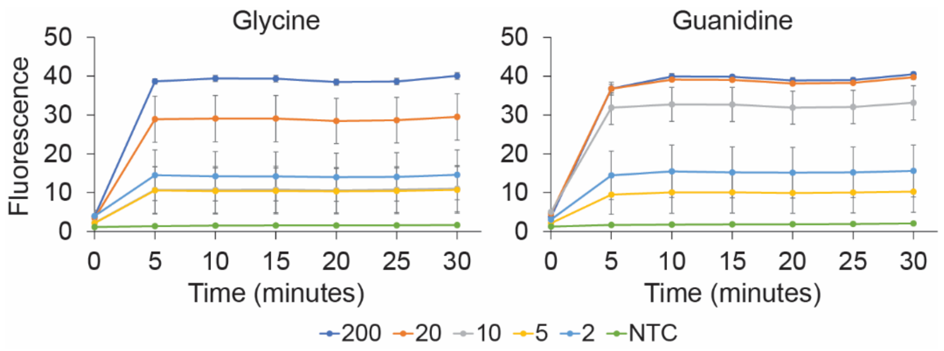

Supplementary Fig. 40 Comparing the assay sensitivity between glycine and guanidine. Different copies of synthetic SARS-CoV-2 RNA fragments were used as input to RT-LAMP, which was performed at 65°C for 15 minutes. Subsequently, 50μl CRISPR reagents were added directly into the LAMP reaction tube and the Cas detection reaction was then carried out at 60°C, with fluorescence measurements taken at 5-minute intervals using a microplate reader. Data represent mean ± s.e.m. (n = 8 biological replicates).

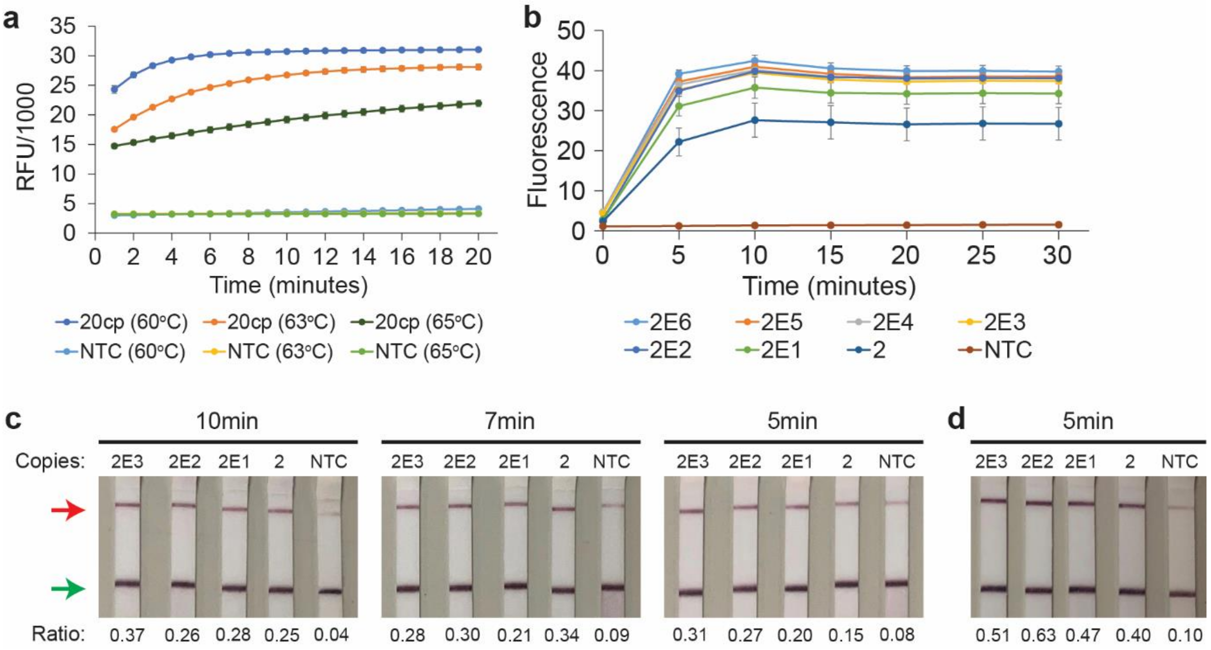

Supplementary Fig. 41 Demonstration of a quasi-one-pot reaction where RT-LAMP and the Cas detection step were performed at similar temperatures.

a Collateral activity of enAsCas12a complexed with both the S2 and S6 4DNA hybrid guides at 60-65°C. Prior to the Cas detection step, RT-LAMP was carried out at 65°C for 15 minutes with 20 copies of synthetic SARS-CoV-2 RNA template as input. Subsequently, 50µl CRISPR reagents in Tango buffer were added directly into the LAMP reaction tube and the Cas detection reaction was then carried out at 60, 63, or 65°C, with fluorescence measurements taken every minute using a real-time instrument.

b Sensitivity of our assay with RT-LAMP performed at a slightly lower temperature of 63°C. Different copies of synthetic SARS-CoV-2 RNA were used as input to the RT-LAMP reaction, which was performed at 63°C for 15 or 17 minutes. Subsequently, 50µl CRISPR reagents in Tango buffer were added directly into the LAMP reaction tube and the Cas detection reaction was then carried out at 60°C, with fluorescence measurements taken at five-minute intervals using a microplate reader. All readings were normalized to NTC at the 0min timepoint. Data represent mean ± s.e.m. (n = 4 biological replicates for 2E3-2E6 copies and n = 15 biological replicates for the other lower copy numbers).

c Detection of SARS-CoV-2 using a lateral flow assay where both RT-LAMP and the Cas detection reaction were performed at 63°C. The enAsCas12a enzyme was complexed with the S2 and S6 4DNA hybrid guides. Different copies of synthetic viral RNA template were used as input to RT-LAMP, which was performed for 22 minutes. Subsequently, the trans-cleavage

reaction was carried out for 5, 7, or 10 minutes before a dipstick was added to each reaction tube. The red arrow indicates the test bands, while the green arrow indicates the control bands. **d** Detection of SARS-CoV-2 using a lateral flow assay where both RT-LAMP and the Cas detection reaction were performed at 60°C. The cleavage reaction was performed for 5 minutes before a dipstick was added to each reaction tube.

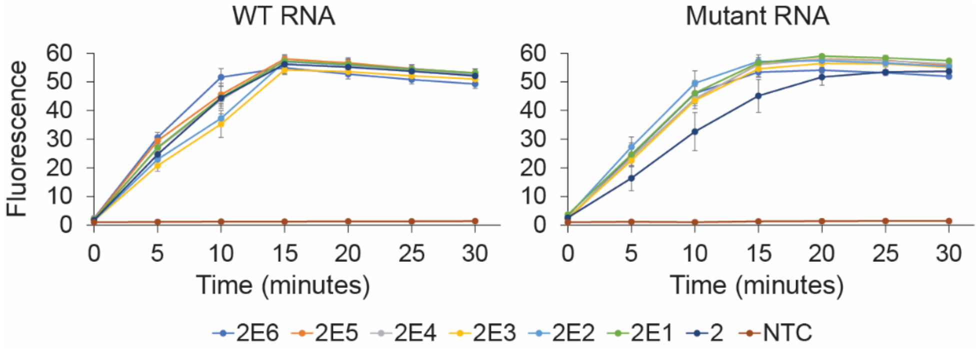

Supplementary Fig. 42 Detection of a real-life mutant SARS-CoV-2 sequence using a quasi-one-pot reaction. The enAsCas12a enzyme was complexed with both the S2 and S6 hybrid guides. Different copies of synthetic SARS-CoV-2 RNA fragments (see legend) were used as input. The fluorescence measurements were taken at 5-minute intervals using a microplate reader. All readings were normalized to NTC at the start of the experiment. The analytical LoD for the wildtype template and the S254F N234N double mutant template appeared to be similar. Data represent mean ± s.e.m. (n = 3 biological replicates).

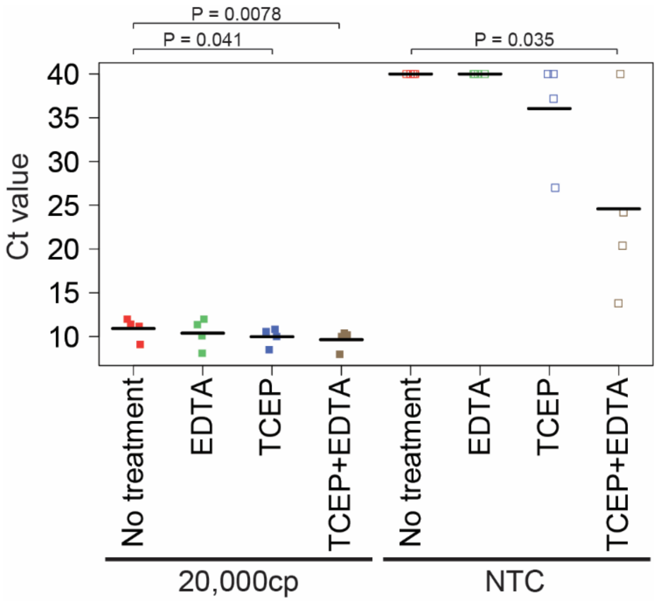

Supplementary Fig. 43 Impact of TCEP and EDTA on RT-LAMP. The isothermal amplification reaction was performed at 65°C for 15 minutes with our full set of primers (including the swarm primers and the truncated primers) in the presence or absence of 2.5mM TCEP or 1mM EDTA. 20,000 copies of synthetic SARS-CoV-2 RNA were used as input. Addition of TCEP and EDTA significantly improved the kinetics of RT-LAMP, but it also caused non-specific amplification. Data represent mean ± s.e.m. (n = 4 biological replicates). P values were calculated using one-sided Student's t-test.

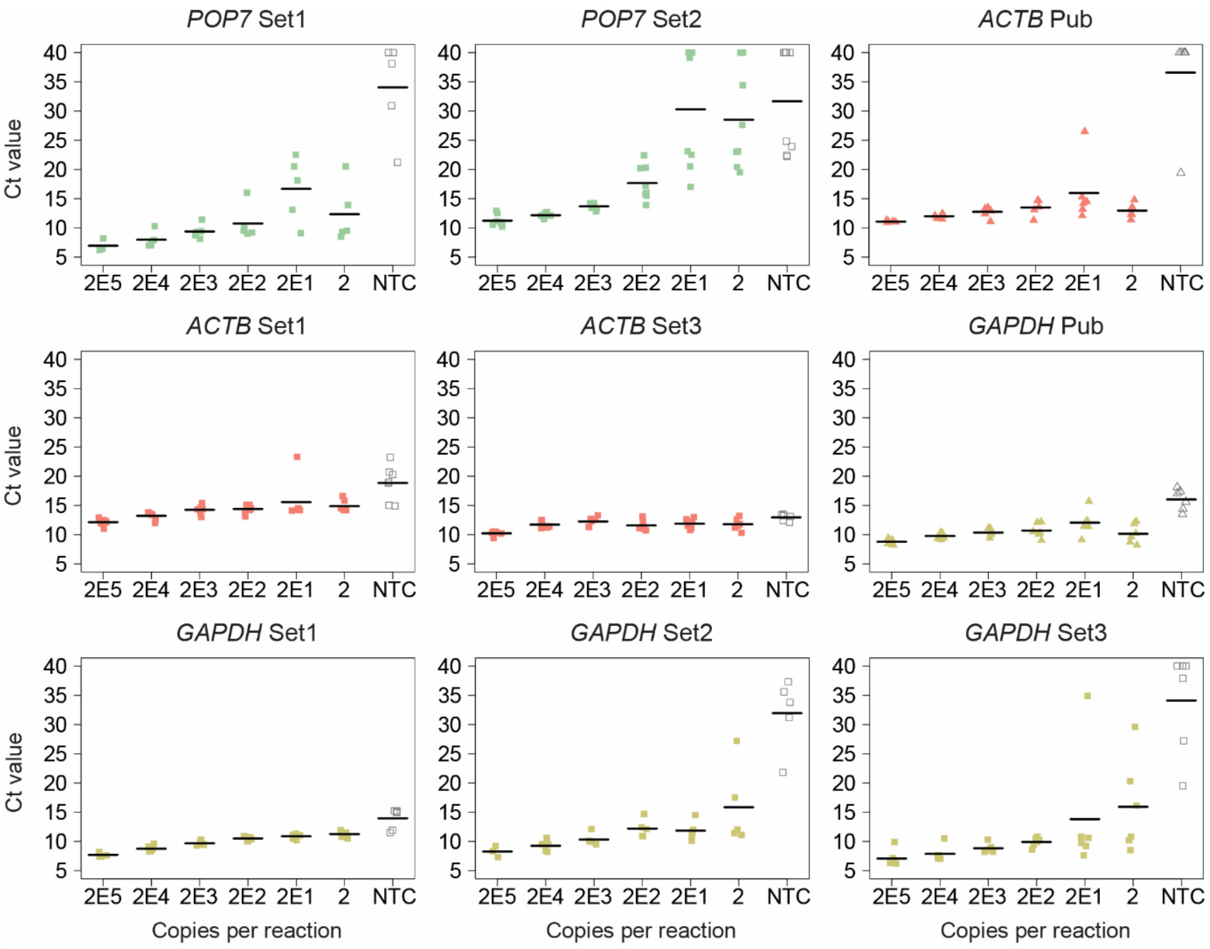

Supplementary Fig. 44 Strip chart showing the effect of different sets of human primers targeting *POP7*, *ACTB*, or *GAPDH* on isothermal amplification of the SARS-CoV-2 S-gene. Different copies of synthetic SARS-CoV-2 RNA were used as sample input to RT-LAMP, which was performed at 65°C over 40 minutes in a real-time instrument. The black horizontal bars among the data points in the strip chart represent the mean (n = 3 [*POP7* Set1 2E5, *GAPDH* Set2 2E5], 5 [*POP7* Set1 all except 2E5, *GAPDH* Set2 all except 2E5], 6 [*ACTB* Pub and Set3, *GAPDH* Pub and Set1+3], 7 [*ACTB* Set1], or 8 [*POP7* Set2] biological replicates).

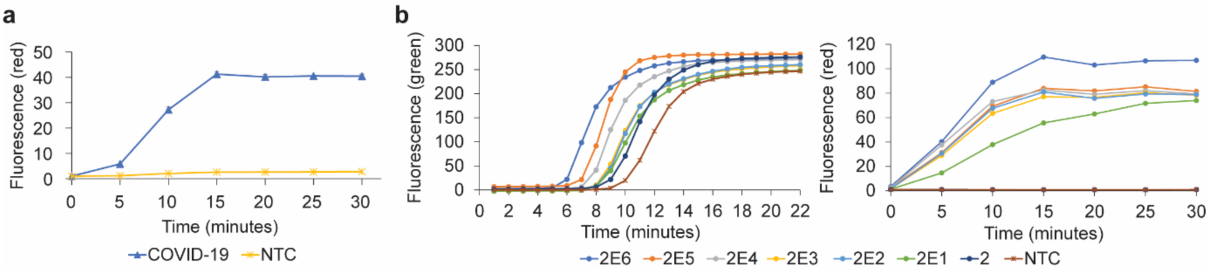

Supplementary Fig. 45 Trans-cleavage assays with a Cy5-quencher reporter.

a Time courses of the fluorescence intensity in our trans-cleavage assays for enAsCas12a complexed with the S2 gRNA. The assays were performed at 60°C and 2E11 copies of DNA template were used as input. All readings were normalized to NTC at the 0min timepoint. Data represent mean ± s.e.m. (n = 3 biological replicates).

b Analytical LoD of our prototype VaNGuard assay with a human internal control. Different copies of synthetic SARS-CoV-2 RNA were spiked into heat-treated healthy donor saliva before being used as input to RT-LAMP, whose reaction mix contained a generic DNA-binding dye (such as SYBR Green or EvaGreen), enabling green fluorescence measurements to be taken every minute (left panel). After 22 minutes of RT-LAMP reaction, 50µl CRISPR reagents containing the Cy5-quencher reporter in Tango buffer were added directly into the LAMP reaction tube. Red fluorescence measurements were then taken every 5 minutes (right panel). Here, the enAsCas12a enzyme was complexed with both the S2 and S6 hybrid guides.

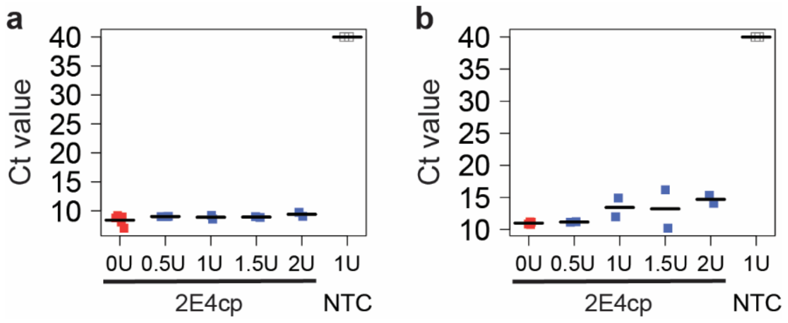

Supplementary Fig. 46 Effect of pyrophosphatase on RT-LAMP.

a We performed isothermal amplification using a WarmStart LAMP Kit from New England Biolabs together with variable amounts (0-2U) of pyrophosphatase. 2E4 copies of synthetic SARS-CoV-2 RNA were used as sample input to RT-LAMP, which was performed at 65°C over 40 minutes in a real-time instrument. The black horizontal bars among the data points in the strip chart represent the mean (n = 2 [1-2U, NTC], 3 [0.5U], or 5 [0U] biological replicates).

b Since the master mix from the WarmStart LAMP Kit contained some unknown components, we also performed isothermal amplification using Bst2.0 in a chemically defined buffer (20mM Tris-HCl, 10mM $(NH_4)_2SO_4$, 50mM KCl, 8mM $MgSO_4$, and 0.1% Tween) together with variable amounts (0-2U) of pyrophosphatase. The black horizontal bars among the data points in the strip chart represent the mean (n = 2 [0.5-2U] or 4 [0U, NTC] biological replicates).

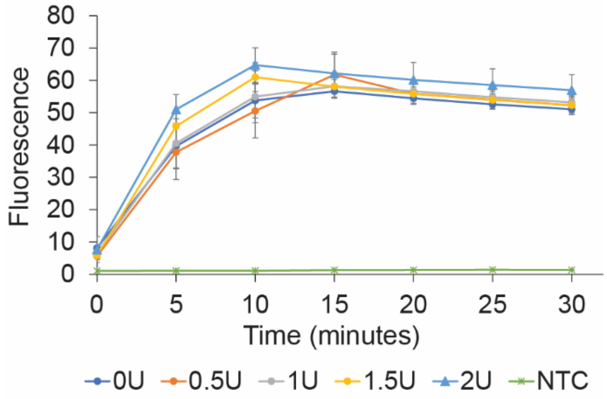

Supplementary Fig. 47 Effect of pyrophosphatase on the CRISPR module. 20 copies of synthetic SARS-CoV-2 RNA were used as input to the quasi-one-pot reaction with different amounts (0-2U) of pyrophosphatase added during the Cas detection step. Fluorescence measurements were taken at 5-minute intervals of the trans-cleavage reaction using a microplate reader. Data represent mean ± s.e.m. (n = 4 biological replicates).

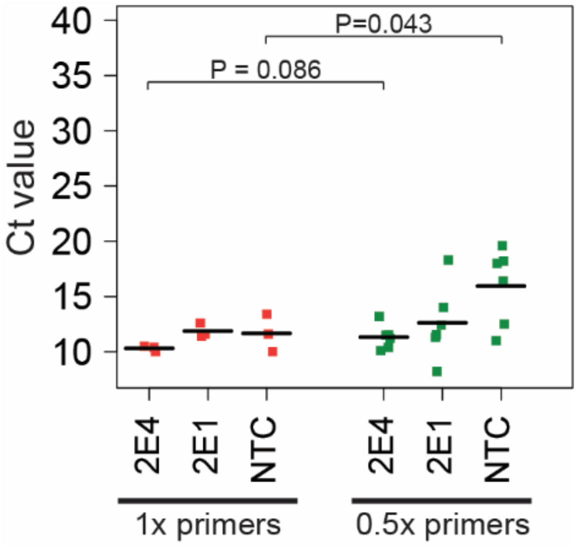

Supplementary Fig. 48 Effect of halving the amount of human LAMP primers on amplification efficiency. 2E1 or 2E4 copies of synthetic SARS-CoV-2 RNA spiked into heat-treated saliva were used as sample input to RT-LAMP, whose reaction mix contained a green DNA-binding dye, the human LAMP primers, and the SARS-CoV-2 LAMP primers. The black horizontal bars among the data points in the strip chart represent the mean (n = 3 biological replicates, with each biological replicate of 0.5x primers performed in two technical replicates). P values were calculated using one-sided Student's t-test.

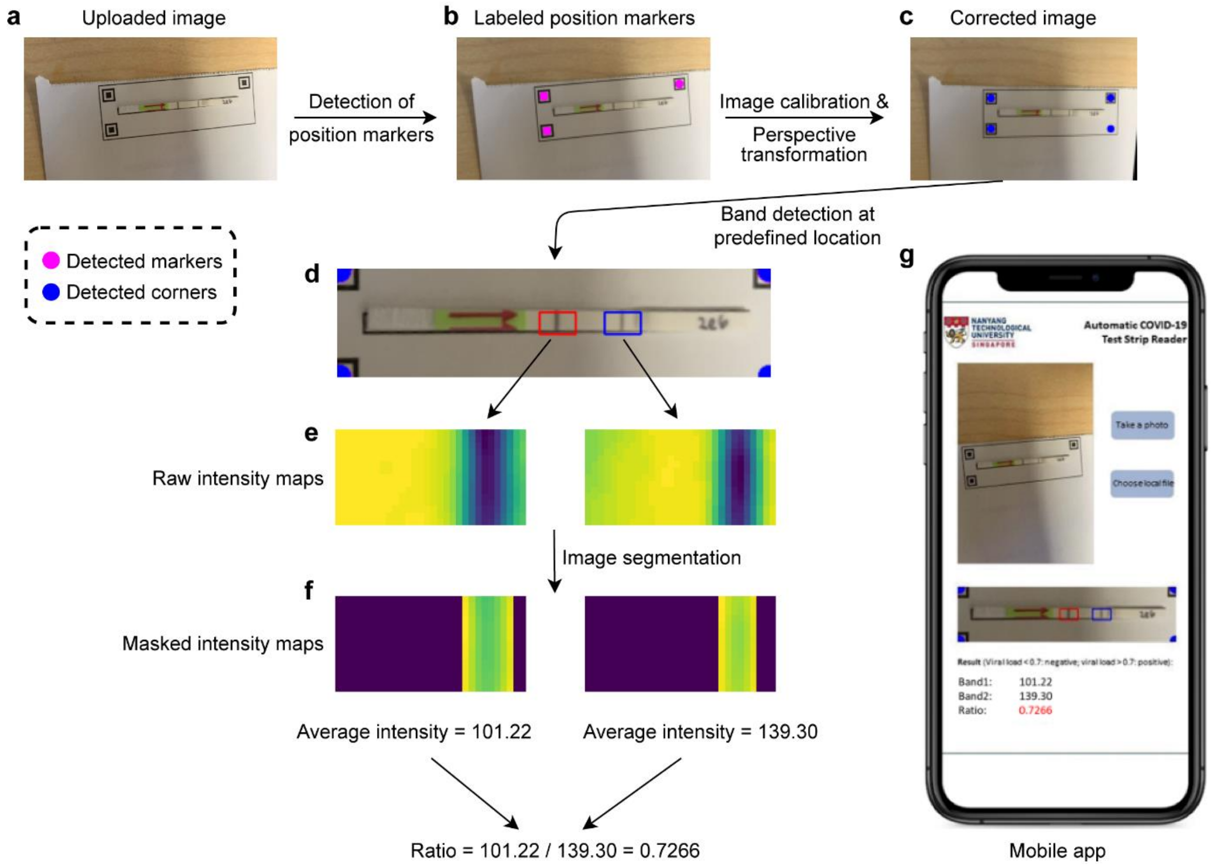

Supplementary Fig. 49 Development of a mobile app to interpret dipsticks.

a Image of an exemplary test strip or dipstick placed on the position template. Here, the image is taken from a random direction under random light conditions.

b The position markers are detected and marked with pink dots. There are three position markers on the template, with each being a square box with a solid square placed at the center of it.

c The corners of the position template are detected and marked with blue dots. Then the image is calibrated and the perspective distortion is corrected according to the detected corners.

d The region bounded by the four detected corners is cropped from the corrected image and resized into a predefined size. The bands are then located from predefined bounding boxes.

e The grayscale intensity maps are extracted from the predefined locations of the resized image.

f Since there will be some background, the selected regions are segmented with Otsu's method. The segments with lower intensities belong to the test and control bands. We then calculate the average intensity of each band from these segments only.

g An image of our mobile app showing the test result.

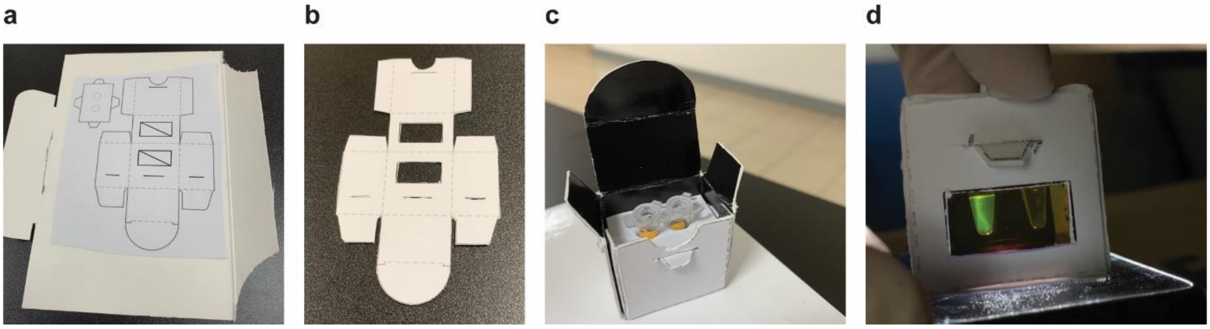

Supplementary Fig. 50 A cheap do-it-yourself (DIY) device from commonly found items to visualize fluorescence test results.

a We created a design on computer, printed it out, and pasted the paper on a piece of discarded cardboard (such as those that came with pipet tip boxes or Eppendorf tubes).

b We cut the cardboard according to our design, including two windows and some slits.

c We folded the cardboard into a box and slotted the tube holder into the box. In addition, we bought colour filter papers from an art and craft shop and pasted them over the windows.

d As a demonstration, we inserted two samples processed by the VaNGuard test into our DIY device and used our mobile phone to shine light through a window into the box. The left tube contained 20 copies of synthetic viral RNA, while the right tube was a no-template control.

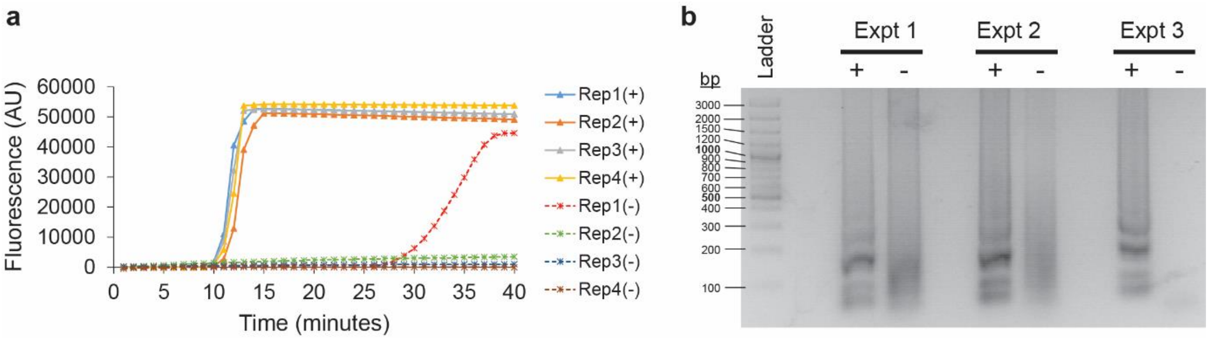

Supplementary Fig. 51 Spurious amplification in LAMP.

a Exemplary fluorescence curves from a real-time instrument for RT-LAMP experiments. Four replicates are shown, where + indicates 20,000 copies of RNA have been added and – indicates NTC (no template control). While the majority of NTC setups showed no amplification, some of the NTC reactions gave late amplifications (high Ct values).

b Gel electrophoresis of LAMP products. In the first two experiments, the NTC reactions showed late amplifications in the real-time instrument, while for the third experiment, the NTC reaction did not give any amplification after 40 cycles (minutes). We then subjected the different samples to gel electrophoresis. As shown in the gel image, products from all the + reactions appear as staggered bands, which are characteristic of successful LAMP. In contrast, products from the first two – reactions appear as smears on the agarose gel, indicating that they are a result of spurious amplification. The three RT-LAMP experiments shown are independent of one another. Overall, we observed that around 10% of the NTCs (7 out of 69 replicates) showed late amplification for our set of S-gene LAMP primers.
