# Supplementary material for: An engineered CRISPR-Cas12a variant and DNA-RNA hybrid guides enable robust and rapid COVID-19 testing
Source: Nat Commun. 2021 Mar 19;12:1739. doi: 10.1038/s41467-021-21996-6 (PMC7979722; doi:10.1038/s41467-021-21996-6)
Supplement: Supplementary file 3 — Reporting Summary [file 41467_2021_21996_MOESM3_ESM.pdf]

nature research

Corresponding author(s): Meng How Tan

Last updated by author(s): Feb 17, 2021

Reporting Summary

Nature Research wishes to improve the reproducibility of the work that we publish. This form provides structure for consistency and transparency in reporting. For further information on Nature Research policies, see our Editorial Policies and the Editorial Policy Checklist.

Statistics

For all statistical analyses, confirm that the following items are present in the figure legend, table legend, main text, or Methods section.

n/a	Confirmed	
☐	☒	The exact sample size (*n*) for each experimental group/condition, given as a discrete number and unit of measurement
☐	☒	A statement on whether measurements were taken from distinct samples or whether the same sample was measured repeatedly
☐	☒	The statistical test(s) used AND whether they are one- or two-sided<br>*Only common tests should be described solely by name; describe more complex techniques in the Methods section.*
☒	☐	A description of all covariates tested
☒	☐	A description of any assumptions or corrections, such as tests of normality and adjustment for multiple comparisons
☐	☒	A full description of the statistical parameters including central tendency (e.g. means) or other basic estimates (e.g. regression coefficient) AND variation (e.g. standard deviation) or associated estimates of uncertainty (e.g. confidence intervals)
☐	☒	For null hypothesis testing, the test statistic (e.g. *F*, *t*, *r*) with confidence intervals, effect sizes, degrees of freedom and *P* value noted<br>*Give P values as exact values whenever suitable.*
☒	☐	For Bayesian analysis, information on the choice of priors and Markov chain Monte Carlo settings
☒	☐	For hierarchical and complex designs, identification of the appropriate level for tests and full reporting of outcomes
☒	☐	Estimates of effect sizes (e.g. Cohen's *d*, Pearson's *r*), indicating how they were calculated

Our web collection on statistics for biologists contains articles on many of the points above.

Software and code

Policy information about availability of computer code

Data collection	A microplate reader was used to collect fluorescence values. A real-time instrument was used to monitor fluorescence and generate Ct values.
Data analysis	Microsoft Excel (Office Professional Plus 2016) and R (v4.0.1) were used to analyze the data. MUSCLE (https://www.ebi.ac.uk/Tools/msa/muscle/) was used to align the genomes of different coronaviruses. BLASTn (https://blast.ncbi.nlm.nih.gov/Blast.cgi) was used to check the specificity of potential gRNAs.

For manuscripts utilizing custom algorithms or software that are central to the research but not yet described in published literature, software must be made available to editors and reviewers. We strongly encourage code deposition in a community repository (e.g. GitHub). See the Nature Research guidelines for submitting code & software for further information.

Data

Policy information about availability of data

All manuscripts must include a data availability statement. This statement should provide the following information, where applicable:

- Accession codes, unique identifiers, or web links for publicly available datasets
- A list of figures that have associated raw data
- A description of any restrictions on data availability

The source data underlying Figs. 1-10 are provided as a Source Data file. Whole genomes of SARS-CoV-2, SARS-CoV, MERS-CoV, CoV OC43, CoV 229E, CoV NL63, and CoV HKU1 are available in GenBank under the accession numbers MN908947.3, NC_004718.3, NC_019843.3, NC_006213.1, NC_002645.1, JX504050.1, and KF686346.1 respectively. Other datasets generated during the current study are available from the corresponding author on reasonable request.

Field-specific reporting

Please select the one below that is the best fit for your research. If you are not sure, read the appropriate sections before making your selection.

- [x] Life sciences
- [ ] Behavioural & social sciences
- [ ] Ecological, evolutionary & environmental sciences

For a reference copy of the document with all sections, see nature.com/documents/nr-reporting-summary-flat.pdf

Life sciences study design

All studies must disclose on these points even when the disclosure is negative.

Sample size	No specific sample-size calculations were carried out. In general, for each set of experiments, at least three biological replicates were conducted, which is standard procedure for molecular biology experiments. We performed sufficient replicates to observe clear trends.
Data exclusions	No experimental data were excluded.
Replication	In general, each set of experiments was repeated at least three times. All attempts at replication were successful. If possible, orthogonal methods were also used to verify the findings (e.g. a microplate reader and dipsticks were independently used to confirm the results).
Randomization	Clinical samples (purified RNAs and NP swabs) were randomly picked to evaluate the VaNGuard assay. Other than this, randomization is not applicable to the rest of our study as there were no other test samples to be assigned to experimental groups.
Blinding	Clinical validations with purified RNAs were performed entirely by hospital staff in the absence of the scientists who developed the assay. Nevertheless, all investigators were not blinded during all experiments in the study.

Reporting for specific materials, systems and methods

We require information from authors about some types of materials, experimental systems and methods used in many studies. Here, indicate whether each material, system or method listed is relevant to your study. If you are not sure if a list item applies to your research, read the appropriate section before selecting a response.

Materials & experimental systems

n/a	Involved in the study
[x]	[ ] Antibodies
[x]	[ ] Eukaryotic cell lines
[x]	[ ] Palaeontology and archaeology
[x]	[ ] Animals and other organisms
[x]	[ ] Human research participants
[x]	[ ] Clinical data
[x]	[ ] Dual use research of concern

Methods

n/a	Involved in the study
[x]	[ ] ChIP-seq
[x]	[ ] Flow cytometry
[x]	[ ] MRI-based neuroimaging
